# Candidate molecular endotypes and dynamic risk prediction in anti-MDA5-positive dermatomyositis-associated interstitial lung disease: a systematic scoping review

**DOI:** 10.3389/fimmu.2026.1979138

**Published:** 2026-10-09

**Authors:** Zhiwei Zuo, Jianguo Yang, Jianbin Li, Renhe Li, Wei Liu

**Affiliations:** 1 First Teaching Hospital of Tianjin University of Traditional Chinese Medicine, Tianjin, China; 2 National Clinical Research Center for Chinese Medicine, Tianjin, China

**Keywords:** anti-MDA5, dermatomyositis, dynamic prediction, interstitial lung disease, machine learning, molecular endotype, multi-omics, radiomics

## Abstract

**Background:**

Anti-melanoma differentiation-associated protein 5 antibody-positive dermatomyositis (anti-MDA5+ DM) spans pulmonary states from no detectable interstitial lung disease (ILD) to rapidly progressive ILD (RP-ILD). We mapped molecular programs and prediction evidence to determine how recurrent biological states may support dynamic stratification.

**Methods:**

PubMed, Web of Science, Ovid and Embase were searched from inception to 22 August 2026. Two reviewers independently screened records and extracted data. Reporting followed PRISMA-ScR; evidence was synthesized across molecular and prediction domains.

**Results:**

Of 4, 166 records, 2, 317 remained after deduplication, 349 underwent full-text assessment, and 95 sources (80 primary studies and 15 syntheses) were included. Type I/III interferon signaling was the most reproducible program. Myeloid inflammatory-remodeling, adaptive activation with lymphocyte or natural-killer-cell loss, vascular-coagulation injury, and immunometabolic disturbance formed additional overlapping axes aligned with pulmonary-state transitions. Clinical and radiomic models showed strong short-term discrimination; harmonized landmarks, calibration, geographic validation, and decision-timed molecular sampling would strengthen clinical use.

**Conclusion:**

The evidence supports an integrated map linking recurrent molecular programs to dynamic pulmonary risk. A calibrated clinical–imaging backbone, augmented by parsimonious molecular modules with incremental value and updated at prespecified landmarks, offers a practical framework for prospective endotype validation and treatment-aware prediction in anti-MDA5+ DM-ILD.

**Systematic review registration:**

https://doi.org/10.37766/inplasy2026.10.0018, identifier INPLASY2026100018.

## 1 Introduction

Dermatomyositis is a systemic autoimmune disease with substantial clinical heterogeneity beyond muscle and skin. Myositis-specific autoantibodies delineate syndromes with distinct organ risks; antibodies to melanoma differentiation-associated protein 5 (MDA5), the cytosolic RNA sensor encoded by IFIH1, identify one of the most consequential groups (1–3). First described as reactivity to a 140-kDa protein in clinically amyopathic dermatomyositis, anti-MDA5 was subsequently linked to MDA5 and rapidly progressive interstitial lung disease (RP-ILD) (4, 5). Vasculopathic skin disease, palmar papules, arthritis, fever, and limited muscle enzyme elevation are frequent but variable (6–9).

Pulmonary trajectories range from no detectable ILD to treatment-responsive disease and fulminant pneumonitis despite intensive immunosuppression (2, 7, 10, 11). Ferritin, C-reactive protein (CRP), lactate dehydrogenase (LDH), lymphocyte count, anti-Ro52 antibodies, oxygenation, pulmonary function, and high-resolution computed tomography (HRCT) carry prognostic information (12–15). Because similar changes accompany autoimmune injury, infection, or both, severity markers require biological and temporal context.

Phenotype and endotype describe different levels of disease. A phenotype is observable, such as RP-ILD or an HRCT pattern; an endotype is a biologically coherent state defined by a proximal mechanism and linked to outcome or treatment response. Recurrent interferon-stimulated gene expression supports an interferon-dominant state, but treatment selection requires evidence of differential benefit. A useful endotype must be reproducible, measurable at the decision point, and informative beyond established severity markers (16).

Evidence across scales now permits such distinctions to be examined. Genetics and environmental studies implicate antigen presentation, RNA sensing, and antiviral exposure (17–19). Bulk and single-cell analyses repeatedly identify interferon programs (20, 21); proteomics adds myeloid, complement, coagulation, and epithelial-injury signals (22); and early metabolomics implicates tryptophan and central-carbon pathways (23). Clinical clustering, risk scores, machine learning, and radiomics have developed in parallel (8, 24–27).

Recent reviews address anti-MDA5 biology, management, viral triggers, macrophage involvement, conventional risk factors, and the broader use of multi-omics and artificial intelligence in autoimmune disease (2, 3, 6, 13, 28–34). We focus on the translational link between molecular states and future pulmonary transitions. The review maps molecular evidence, evaluates candidate endotypes against explicit validation criteria, examines current prediction targets and time horizons, and proposes a treatment-aware longitudinal framework.

### 1.1 Review scope and terminology

RP-ILD generally denotes new or worsening respiratory symptoms, hypoxemia, and/or radiographic progression within one to three months, although time zero varies among symptom onset, ILD diagnosis, enrollment, and baseline imaging (10, 14). We retained each source definition and landmark.

Here, ‘multi-omics integration’ means joint analysis of at least two molecular layers in the same participants; ‘cross-omics convergence’ denotes concordant signals assembled across separate cohorts.

## 2 Methods

### 2.1 Review design and eligibility

This systematic scoping review followed PRISMA-ScR, with study selection reported using the PRISMA 2020 flow structure (35–37). The review question was: across anti-MDA5-positive dermatomyositis pulmonary states, which recurrent molecular programs and prediction approaches have been reported, and what evidence supports their use for decision-timed stratification?

Eligibility followed a Population-Concept-Context framework cross-walked to PICOS. The population comprised anti-MDA5-positive dermatomyositis from no detectable ILD to chronic ILD and RP-ILD. Eligible data were genomic, transcriptomic, single-cell, proteomic, metabolomic, clinical, imaging, radiomic, or machine-learning; outcomes included ILD status or progression, RP-ILD, mortality, recovery, relapse, fibrosis, treatment response, and model performance. No intervention or comparator was required; both were charted when treatment effects were reported. Eligible sources were peer-reviewed English-language human observational, patient-derived mechanistic, prediction-model, and focused synthesis reports. We excluded animal-only studies, inadequately reported abstracts, editorials, non-English reports, and reports without relevant anti-MDA5 or pulmonary evidence. English-language eligibility reflected capacity for consistent full-text assessment and charting.

### 2.2 Information sources and search strategy

PubMed, Web of Science, Ovid and Embase were searched from inception to 22 August 2026. Controlled vocabulary and free-text terms covered the anti-MDA5/MDA5 (IFIH1) axis, dermatomyositis, and ILD, including RP-ILD. No study-design, language, or species filter was applied at the search stage; non-English and non-human records were excluded during screening. Complete strategies are provided in **Supplementary Methods 1**.

### 2.3 Study selection and data extraction

After deduplication, two reviewers independently screened titles and abstracts and assessed potentially eligible full texts. Disagreements were resolved by consensus or a third reviewer, and full-text exclusions were recorded. Two reviewers independently extracted study design, population, sample size, data source, platform, sampling landmark, treatment exposure, molecular state or prediction target, pulmonary outcome, validation, and performance; discrepancies were reconciled. Prediction and monitoring studies summarized in **Table 1** were additionally subjected to a structured methodological appraisal informed by PROBAST+AI, focusing, where applicable, on participant and data-source selection, predictor and outcome definition, sample size and event numbers, model development and validation, calibration, and transportability. The appraisal was used to identify key methodological limitations and contextualize reported performance.

**Table 1:** Selected prediction and longitudinal-monitoring studies mapped in the systematic scoping review.

Study/model	Target, horizon, population, n/events, and landmark	Predictors/model	Validation and reporting	Reported performance	Methodological/decision-use consideration
CROSS (25)	Incident RP-ILD within 1 year; n=170 development and n=85 geographic validation; diagnosis/referral landmark	CRP, anti-Ro52, disease duration ≤3 months, and male sex	Geographic validation within Jiangsu; time-dependent discrimination; calibration incompletely reported	Time-dependent AUC >0.80 throughout 1 year; very-high-risk 1-year incidence 71.3% and 85.7%	Dichotomization, referral-time dependence, and limited geographic range
CRAFT (82)	Incident RP-ILD; n=53, 21 events; single-center baseline	CRP/albumin ratio, RDW-CV, fever, and CD3+ T cells	Bootstrap internal validation; calibration and decision curve reported	AUC 0.93 (95% CI 0.86–0.99); sensitivity 0.81; specificity 0.94	Very low event count and no independent validation
FLAW (83)	Incident RP-ILD; multicenter n=116, 47 events; baseline	Fever, LDH, age, and white-cell count	Internal validation subset; calibration and decision analysis not reported	RP-ILD probability was 100% at score 4; validation risks were 0%, 0%, 42.9%, and 75% at scores 0–3	Small validation subset and unestablished geographic transportability
FLAIR (84)	1-year mortality; amyopathic DM-ILD n=207 (not exclusively anti-MDA5+); baseline	Ferritin, LDH, anti-MDA5 grade, HRCT score, and RP-ILD	Internal validation; Brier score reported	1-year Brier score 0.106	Outcome-proximal RP-ILD and broader target population limit preventive use
MCK model/JAMI (85)	All-cause mortality; incident PM/DM-ILD; n=497 derivation and n=111 independent validation; baseline	Anti-MDA5 status, CRP, and KL-6	Multicenter derivation and separate validation cohort	Low (<15%), moderate (15–50%), and high (≥50%) mortality-risk groups; survival separation replicated	Broader PM/DM-ILD population and baseline-only stratification
Gui et al. (86)	1-year mortality; anti-MDA5+ DM-ILD; n=89 discovery and n=38 temporal validation; pretreatment	Age, heliotrope rash, fever, and laboratory score (lymphocytes, CD4+ T cells, CYFRA21-1, oxygenation index, and anti-Ro52)	Temporal validation; calibration and decision curves	AUC 0.915 (95% CI 0.846–0.957) in discovery and 0.971 (95% CI 0.813–0.978) in validation	Single-center temporal validation; treatment-effect analysis was exploratory
Niu et al. (87)	Poor outcome/death through 400 days; anti-MDA5+ DM-ILD n=63, 19 events; baseline	Disease duration, fever, pleural effusion, total CT score, and AST	Retrospective derivation without external validation	Combined AUC 0.954 (95% CI 0.902–1.000); sensitivity 84.2%; specificity 94.6%	Low event count and no independent validation
Ouyang et al. (88)	6-month mortality; n=40 derivation and n=29 external validation; baseline	Fever, ferritin ≥1, 250 μg/L, and positive CEA	External institutional cohort	Mortality in low/moderate/high strata: 0%/11%/85% in derivation and 25%/43%/78% in validation	Small cohorts, case-mix shift, and incomplete probability calibration
FLATCAN (89)	3-, 6-, and 12-month mortality; anti-MDA5+ DM n=621; baseline	Ferritin, LDH, age, CD8+ T cells, CRP, albumin, and NSIP-plus-OP; Cox model	Cross-validation and internal/temporal splits	C-index 0.815; AUC 0.895, 0.855, and 0.850 at 3, 6, and 12 months	No geographic external validation or treatment-effect modeling
Li et al. multicenter model (26)	3-month mortality; hospitalized DM-ILD n=509 across six hospitals; admission	Eight routine predictors; logistic regression selected over five flexible algorithms	Internal test and two external hospital cohorts; calibration, decision curves, and SHAP	AUC 0.866 internally; 0.836 and 0.915 externally	RP-ILD as a predictor limits pre-emption; retrospective Chinese treatment context
Xu et al. radiomics (90)	6-month all-cause mortality; n=228 across training, test, temporal, and external sets; admission/baseline CT	CT radiomics score, FVC%, and age	Test, temporal, and small external cohorts	C-index 0.88, 0.88, 0.83, and 0.84; external Brier score 0.11	Scanner, reconstruction, and segmentation transportability
Li et al. radiomic nomogram (91)	Incident RP-ILD; n=103; baseline CT and clinical assessment	Disease duration, dyspnea, and three CT radiomic features	Train/test split	Test AUC 0.812	Marked optimism from training to test and no geographic external calibration
He et al. radiomics (27)	Incident RP-ILD; n=119 training, n=41 internal validation, and n=29 external validation; baseline HRCT	Bilateral-lung radiomics score, consolidation score, LDH, and infection	External institution	Combined AUC 0.882 internally and 0.916 externally	Small external cohort; infection affects both texture and outcome
Zhang et al. visual HRCT (92)	Incident RP-ILD; single-center n=67; baseline HRCT	Semiquantitative HRCT extent plus clinical variables	Internal validation	AUC 0.842	Accessible and interpretable, but small cohort without external calibration
Chen et al. antibody trajectory (50)	Long-term control among n=310 six-month survivors; baseline and 3-month titers	Latent anti-MDA5 titer trajectories; ≥33% reduction at 3 months	Internal trajectory derivation; no external validation	AUC 0.86 for fast versus slow seroconversion	Survivor conditioning excludes the earliest fatal-risk period
Kitahara et al. IFN-λ3 (80)	Death or relapse within 1 year; n=24, 8 events (6 deaths and 2 relapses); diagnosis and 1 month	Failure of serum IFN-λ3 to decline by 1 month	Internal longitudinal association; no external threshold validation	Associated with death or relapse	Small cohort and treatment-confounded kinetics
Zhang et al. ferritin trajectory (106)	Mortality; longitudinal anti-MDA5+ DM cohort; serial ferritin landmark	Serum ferritin trajectory groups	Internal trajectory modeling; no external validation	Trajectories separated mortality risk	Measurement intensity, treatment, and informative missingness may bias trajectories

Event counts are shown where reported; performance values are not directly comparable across populations, landmarks, outcomes, horizons, and validation designs. AST, aspartate aminotransferase; AUC, area under the receiver-operating-characteristic curve; CEA, carcinoembryonic antigen; CI, confidence interval; C-index, concordance index; CRP, C-reactive protein; CT, computed tomography; CYFRA21-1, cytokeratin 19 fragment; DM, dermatomyositis; FVC, forced vital capacity; HRCT, high-resolution computed tomography; ILD, interstitial lung disease; KL-6, Krebs von den Lungen-6; LDH, lactate dehydrogenase; MDA5, melanoma differentiation-associated protein 5; NSIP, nonspecific interstitial pneumonia; OP, organizing pneumonia; PM, polymyositis; RDW-CV, red-cell distribution-width coefficient of variation; RP-ILD, rapidly progressive interstitial lung disease; SHAP, SHapley additive explanations.

## 3 Results

### 3.1 Evidence selection and source characteristics

The searches returned 4, 166 records: 877 from PubMed/MEDLINE, 814 from Web of Science Core Collection, 612 from Ovid MEDLINE, and 1, 863 from Embase. Removing 1, 849 duplicates left 2, 317 records; 1, 968 were excluded during title and abstract screening. All 349 reports were retrieved for full-text assessment. Exclusions were overlapping data (n = 86), sources outside the prespecified domains (n = 58), ineligible designs (n = 69), ineligible populations or anti-MDA5 status (n = 14), and no eligible pulmonary, molecular, imaging, or prediction outcome (n = 27). Ninety-five sources entered the synthesis (**Figure 1**).

**Figure 1:**
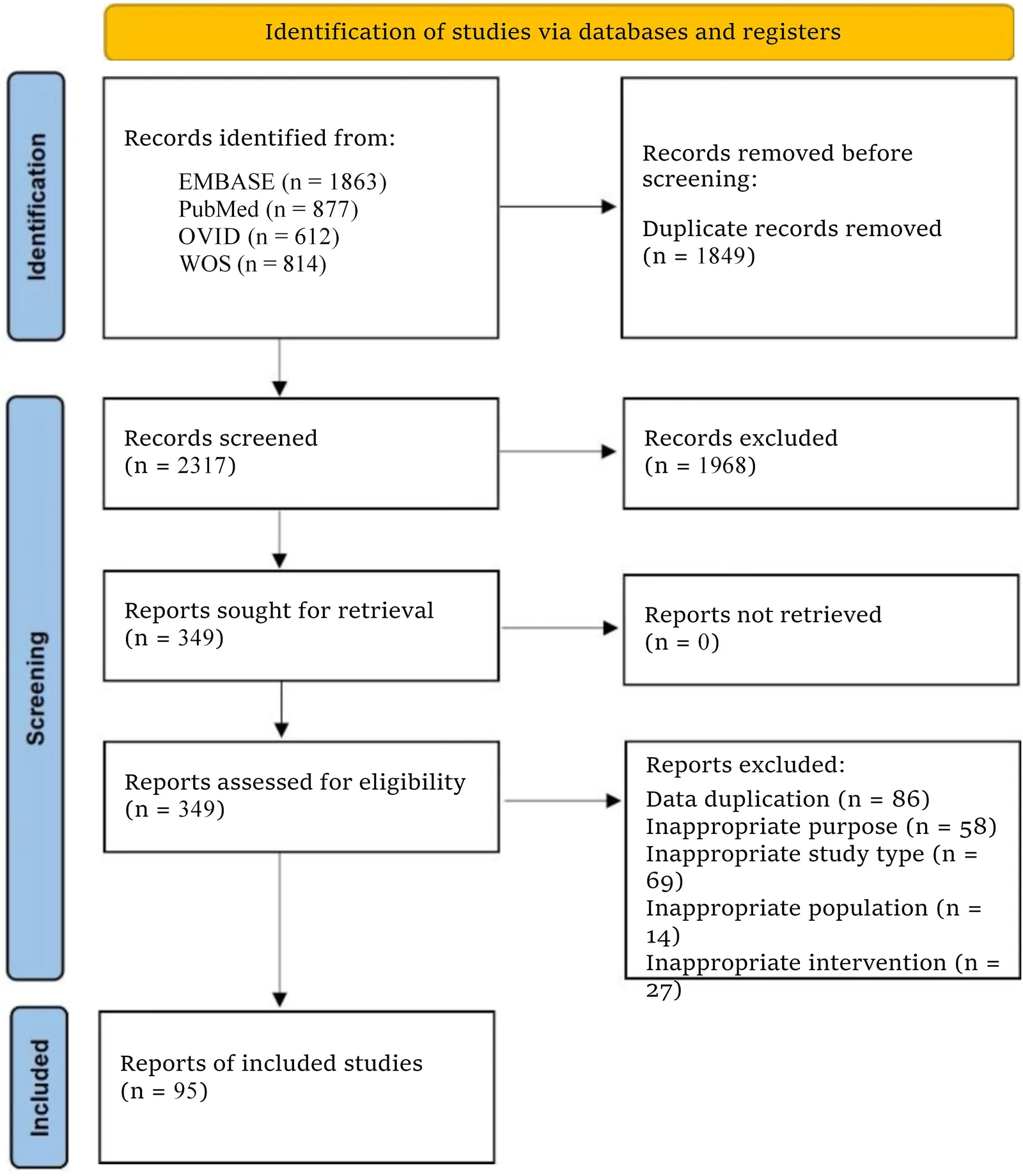
PRISMA-ScR flow diagram. Searches identified 4, 166 records; 2, 317 remained after deduplication, 349 reports underwent full-text assessment, and 95 sources were included.

The 95 sources encompassed phenotype and outcome cohorts, genetics, patient-derived mechanistic studies, bulk and single-cell transcriptomics, proteomics, metabolomics, biomarker trajectories, prediction models, radiomics, therapeutic evidence, and focused syntheses. Sources could inform more than one domain. **Supplementary Table 1** lists the complete evidence inventory and interpretive role. Mechanistic evidence was commonly exploratory and single-center, whereas independent or temporal validation was concentrated in a subset of clinical and radiomic models; source-level design and reporting considerations are summarized in **Supplementary Table 1**.

The corpus integrates observational, mechanistic, and prediction research, with particularly rich evidence from East Asian referral centers. **Tables 1**–**3** summarize sampling context, temporal landmarks, and validation features.

**Table 2:** Selected molecular evidence sources mapped in the systematic scoping review.

Evidence source(s)	Population, setting, and sample size	Design, biospecimen, and sampling landmark	Principal molecular signal	Pulmonary or clinical relevance	Interpretive consideration
Gono et al. (2012); Chen et al. (2017) (17, 51)	Gono: Japanese anti-MDA5+ DM cohort; Chen: 70 PM, 104 DM, and 400 HCs (Han Chinese)	Cross-sectional HLA-DRB1 genotyping; germline DNA	Population-specific HLA-DRB1 susceptibility alleles	Anti-MDA5 autoantibody susceptibility	Ancestry-specific frequencies, modest samples, and limited independent replication
Kochi et al. (2018); Guo et al. (2023) (18, 52)	Kochi: CADM n=576; Guo: anti-MDA5+ DM n=254 (190 CADM), anti-MDA5− DM n=53, antisynthetase syndrome n=72, and HCs n=192	Germline genotyping, eQTL, and functional signaling; PBMC expression and lung tissue in Guo	WDFY4 splice variation and augmented MDA5/NF-κB signaling	CADM susceptibility and RP-ILD association	Population-specific associations; no individual-level prediction or ancestry-diverse validation
Yang et al. (2025); Okamoto et al. (2025) (53, 54)	Yang: 231 anti-MDA5+ DM, 127 anti-MDA5− DM, 1, 468 disease controls, and 1, 027 HCs; Okamoto: 204 DM (174 ILD; 62 anti-MDA5+), six Japanese centers	NGS HLA typing; WES discovery (n=9) followed by Sanger sequencing (n=204); blood DNA	HLA-DQA1*06:01 and candidate IFIH1 variants	Anti-MDA5 susceptibility and prognosis; ILD or acute-onset risk	Exploratory variants require independent ancestry-diverse replication
Qian et al. (2023) (20)	Autoimmune disease n=262 (anti-MDA5+ DM n=61) plus 58 HCs; single center	Baseline PBMC RT-qPCR IFN score; serial in-hospital samples in 16 patients; treatment at sampling varied	High type I IFN activity in most patients	Disease activity and 3-month survival	Single center; 13 deaths; no external threshold validation; treatment confounding
Li et al. (2023); Wang et al. (2023) (58, 59)	Li: RNA-seq in 23 IIM (9 anti-MDA5+) and 8 HCs, with validation cohorts; Wang: RNA-seq in 13 patients and 7 HCs, with longitudinal cohort n=49	PBMC/monocyte transcriptomics and serum IFN scores; onset/relapse and post-treatment samples	Monocyte-centered type I IFN program; nonparallel antibody and IFN kinetics	Subtype distinction, activity, and monitoring	Cell composition, treatment, infection, and sampling-time effects
Fukada et al. (2024); Yoshida et al. (2025); Kitahara et al. (2026) (62, 63, 80)	Fukada: 221 PM/DM-ILD and 38 controls (anti-MDA5+ outcome cohort n=79); Yoshida: anti-MDA5+ DM n=53; Kitahara: anti-MDA5+ DM-ILD n=24	Baseline serum IFN-λ3 or type I/III IFN profiling with derivation/validation; serial post-treatment sampling	Type I/III IFN dysregulation and IFN-λ3 trajectories	Poor outcome and short-term disease control	Assay thresholds, treatment exposure, and timing require external standardization
Ye et al. (2022) (21)	Active anti-MDA5+ DM n=7, paired remission n=3, disease controls n=5, and HCs n=4; survival cohort n=31	Single-cell profiling of circulating B/T cells; exploratory lung dataset from one transplant recipient	Plasmablasts, clonal CD8+ cells, ISG15+ CD8+ state, and metabolic reprogramming	One-year survival and therapeutic hypotheses	Small discovery series; lung component derived from one affected patient
He et al. (2024); Weng et al. (2025) (67, 68)	He: small anti-MDA5+ DM discovery series; Weng: RP-ILD n=4, non-RP-ILD ILD n=3, and HCs n=3	PBMC single-cell RNA sequencing; Weng patients sampled after initial glucocorticoid exposure	IFI27/IFIH1-high monocytes, plasma cells, T/NK-cell depletion, and antiviral activation	RP-ILD and severe immune phenotype	Small discovery cohorts, mixed treatment, and limited mild-disease controls
Shi et al. (2025) (69)	Severe anti-MDA5+ DM-ILD n=5 with paired blood/BALF; orthogonal serum/BALF cohorts	Paired blood/BALF single-cell profiling with flow-cytometric and Luminex validation	Transition from circulating monocytes to inflammatory/profibrotic lung macrophages	Severe alveolar inflammation and remodeling	Very small sample; late-stage lung sampling limits temporal inference
Guo et al. (2024); Zhang et al. (2026); Qian et al. (2026) (41, 42, 72)	Guo: inpatients n=123 (activation-marker subset n=33); Zhang: referral cohort; Qian: n=64 (13 deaths by day 180)	Admission/early blood flow cytometry, immune-cell clustering, and CD4+ memory profiling	Treatment-sensitive immune-cell states and lymphocyte loss	RP-ILD, infection, and early mortality	Cohort-specific clusters lack external assignment rules and longitudinal stability
Qiu et al. (2024) (22)	Discovery: anti-MDA5+ DM n=20 and HCs n=10; validation: RP-ILD n=20, non-RP-ILD n=20, and HCs n=20	Baseline plasma mass-spectrometry discovery with targeted ELISA validation	SPP1 with acute-phase, complement, and coagulation modules	RP-ILD and pulmonary severity	Circulating source and temporal ordering remain uncertain
Hensgens et al. (2022); Zhang et al. (2025) (73, 74)	Hensgens: Dutch CADM n=20 (serum subset n=16) plus 15 HCs; Zhang: IIM n=77 (34 anti-MDA5+) plus 53 HCs	Serum proximity-extension/Olink inflammatory proteomics; active-disease sampling	IL-6, IL-8, IL-18-related proteins, CX3CL1, KRT19, PD-L1, and broad inflammation	RP-ILD phenotype and anti-MDA5 classification	Small disease-specific samples and overlap with infection or critical illness
Liang et al. (2021) (75)	Anti-MDA5+ DM-ILD cohort	Serum galectin-9 measurement with mechanistic assessment	Interferon-associated galectin-9 elevation	Pulmonary severity	Association does not establish source, specificity, or incremental value
Liu et al. (2026) (23)	RP-ILD n=25, chronic ILD n=25, no ILD n=24, and HCs n=41	Cross-sectional plasma metabolomics	Tryptophan-kynurenine and broader amino-acid disturbance	Pulmonary phenotype separation	Diet, organ function, microbiome, hypoxia, and reverse causation
Li et al. (2025) (76)	Active untreated IIM n=183 plus 104 HCs; discovery included anti-MDA5+ DM n=67; independent validation cohort	Baseline plasma and blood-cell untargeted/targeted metabolomics	Citrate and dimethylglycine with central-carbon reprogramming	Diagnosis, RP-ILD, and mortality	No independent prognostic validation or flux confirmation

Selected sources anchor the cross-platform synthesis. BALF, bronchoalveolar lavage fluid; CADM, clinically amyopathic dermatomyositis; DM, dermatomyositis; ELISA, enzyme-linked immunosorbent assay; eQTL, expression quantitative trait locus; HC, healthy control; HLA, human leukocyte antigen; IFN, interferon; IIM, idiopathic inflammatory myopathy; IL, interleukin; ILD, interstitial lung disease; MDA5, melanoma differentiation-associated protein 5; NGS, next-generation sequencing; NF-κB, nuclear factor kappa B; NK, natural killer; PBMC, peripheral blood mononuclear cell; PM, polymyositis; RNA-seq, RNA sequencing; RP-ILD, rapidly progressive interstitial lung disease; RT-qPCR, reverse-transcription quantitative polymerase chain reaction; SPP1, secreted phosphoprotein 1; WES, whole-exome sequencing.

**Table 3:** Candidate molecular axes, current evidence, and decisive validation requirements.

Candidate axis	Evidence sources/material	Biological program	Candidate measurement	Evidence state	Major uncertainty	Decisive validation test
Interferon-antiviral	Germline DNA, blood transcriptomes, serum cytokines, PBMC and lung immune cells (18–21, 53, 54, 58–66, 69, 80)	MDA5–RNA sensing and type I/III IFN programs across monocytes, lymphocytes, epithelium, and macrophages	Locked ISG score; IFN-β/IFN-λ3; CXCL10; cell-resolved module	Repeated across platforms; no treatment-interaction evidence	Infection, cell composition, treatment, and disease-time effects	Pretreatment and days 3–7 sampling; locked assay threshold; external validation; randomized or target-trial treatment interaction
Myeloid inflammatory-remodeling	Blood monocytes, paired blood/BALF single-cell data, and plasma proteomics (22, 31, 69, 73, 74, 81)	Inflammatory-monocyte recruitment and differentiation into inflammatory/profibrotic lung macrophages	Monocyte-state score; SPP1-centered panel; CCL2/CX3CL1; distal ferritin/CD206	Moderate cross-platform support; temporality unresolved	Critical illness and infection can generate the same soluble profile	Show module change before oxygenation/HRCT change and test treatment interaction
Adaptive activation and loss	B/T-cell single-cell data, flow cytometry, clonality, and functional IgG studies (21, 42, 56, 57, 67–72)	Plasmablast and clonal CD8+ expansion with apoptosis, exhaustion, redistribution, or T/NK-cell depletion	BCR/TCR clonality; ISG15+ CD8+ cells; plasmablast and NK/CD4+ memory modules	Moderate; small discovery cohorts	Drug-induced depletion and tissue redistribution mimic disease states	Joint pretreatment clonality, cell-fate, and tissue-location measurement with external assignment
Vascular-coagulation injury	Plasma proteomics, cutaneous phenotype, and limited tissue evidence (22, 73, 74)	Complement, endothelial, platelet/coagulation activation, and immunothrombosis	Complement split products; kininogen 1; endothelial/platelet markers; spatial pathology	Low to moderate; enrichment exceeds tissue proof	Anticoagulation, immobility, infection, and critical illness	Confirm local activation and temporality in matched blood, skin, and lung
Immunometabolic	Plasma and blood-cell metabolomics plus single-cell metabolic programs (21, 23, 76)	Tryptophan-kynurenine, central-carbon, one-carbon, glycolytic, and mitochondrial change	Targeted metabolite ratios or flux-based module	Preliminary	Diet, fasting, microbiome, hypoxia, renal/hepatic function, and handling	Standardized serial sampling; external incremental value; cellular flux validation

Axes are continuous and overlapping. ‘Repeated across platforms’ indicates concordant findings from at least two independent cohorts and two assay modalities or biological materials; ‘moderate’ indicates support from more than one study without full cross-platform replication; ‘preliminary’ indicates discovery-stage evidence. These are mapping categories, not formal certainty ratings. BALF, bronchoalveolar lavage fluid; BCR, B-cell receptor; HRCT, high-resolution computed tomography; IFN, interferon; IgG, immunoglobulin G; ISG, interferon-stimulated gene; MDA5, melanoma differentiation-associated protein 5; NK, natural killer; PBMC, peripheral blood mononuclear cell; RP-ILD, rapidly progressive interstitial lung disease; SPP1, secreted phosphoprotein 1; TCR, T-cell receptor.

### 3.2 Pulmonary trajectories and clinical heterogeneity

#### 3.2.1 Pulmonary course is a dynamic state

The clinically relevant spectrum extends beyond ILD present versus absent. Patients may have no detectable pulmonary involvement, limited abnormalities, chronic or relapsing disease, or RP-ILD with enlarging ground-glass opacity or consolidation, worsening gas exchange, and concentrated early mortality (2, 7, 10–12). These states can change: mild disease may remain stable or accelerate, and RP-ILD survivors may develop fibrosis. Sampling time therefore alters the meaning of a ferritin value, CT pattern, or molecular signature.

Extrapulmonary findings further resolve the syndrome. Skin ulceration, palmar papules, arthritis, fever, and constitutional symptoms may precede lung disease, whereas muscle weakness may be subtle or absent (6, 8). European and North American series have reported more prominent cutaneous and articular disease and fewer fulminant pulmonary events than many East Asian referral cohorts (8, 38), indicating that susceptibility, exposure, referral, assay, and treatment shape organ expression.

Data-driven studies confirm structured heterogeneity. A French cohort of 121 patients identified RP-ILD-, rheumatic-, and vasculopathic-skin-dominant groups (8). Chinese cohorts also resolved three clusters using different variables: mild rash-and-muscle, arthritis-rich, and high-mortality RP-ILD patterns (24), or lymphocyte counts and broader clinical features (39, 40). Flow cytometry added risk patterns (41); six immune-cell proportions separated 123 inpatients by RP-ILD, infection, treatment intensity, and three-month mortality (42). Common definitions and external assignment rules remain essential (43), despite a subsequent three-stratum solution (44).

Repeated three-cluster solutions do not imply the same biology. Clinical analyses emphasized pulmonary, rheumatic, cutaneous, or lymphocyte-related severity, whereas the immune-cell study reflected circulating composition, infection, and treatment exposure (24, 39–44). The studies nevertheless establish reproducible structure and supply variables for harmonized, externally assignable subgroups.

#### 3.2.2 Geography and ascertainment alter the observed syndrome

Transportability is both biological and clinical. In a standardized comparison of 34 Brazilian and 65 Japanese patients, ILD occurred in 50.0% and 98.5% and RP-ILD in 3.3% and 52.3%, respectively (9). Muscle and several cutaneous findings were more frequent in Brazil, whereas pulmonary disease dominated in Japan. A North American cohort documented sustained drug-free remission in a subset (38). During the COVID-19 pandemic, a Yorkshire series reported increased MDA5 autoimmunity and pneumonitis linked to host IFIH1 variation (45).

Ancestry, referral pathways, assays, calendar period, and treatment jointly influence these differences. Transfer therefore requires direct comparison of predictor distributions, baseline risk, and predictor–outcome associations, together with measurement of allele frequencies, infection history, time to specialist care, CT acquisition, antibody assay, and prescribing practice.

#### 3.2.3 Severe inflammation, infection, and treatment are entangled

Infection can mimic or amplify autoimmune lung injury: fever, rising CRP or LDH, lymphopenia, and new CT opacity occur in both. Pneumocystis jirovecii pneumonia and other opportunistic infections arise early during intensive immunosuppression, and a 92-patient cohort associated Pneumocystis infection with higher three-month mortality (46). Because infection also changes transcriptomic, proteomic, and radiomic features, molecular data obtained during respiratory failure require concurrent pathogen assessment.

Treatment is a second time-varying influence. Patients at greatest risk receive earlier intensive therapy, which alters biomarkers and outcomes (47–49). Post-treatment samples characterize a modified state, and later sampling also reflects survival and prior clinical decisions (50). **Figure 2** therefore frames disease as transitions from susceptibility and exposure through immune state and tissue injury, observed incompletely by blood, physiology, and imaging and modified by infection and treatment.

**Figure 2:**
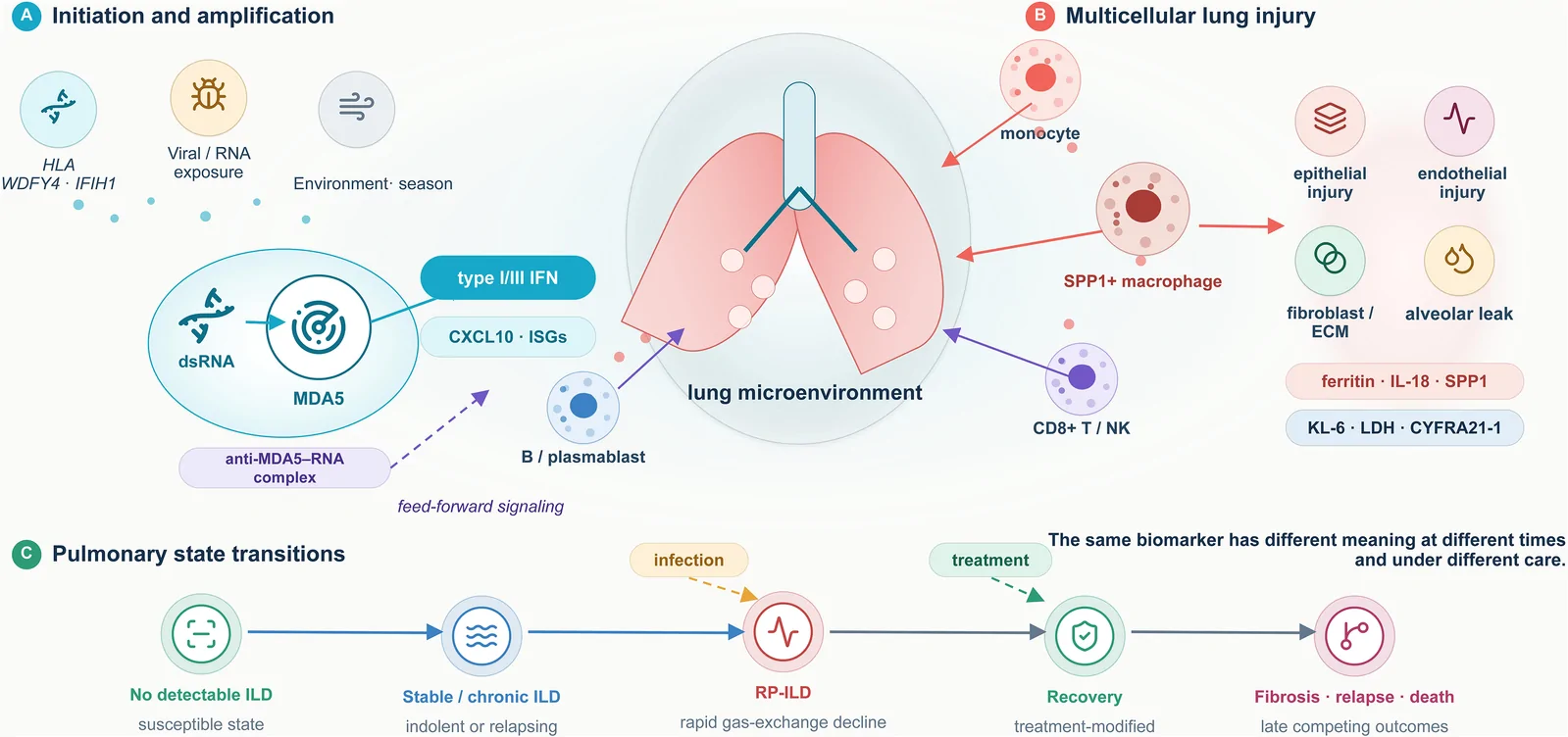
Immune activation, lung injury, and pulmonary transitions in anti-MDA5+ DM-ILD. **(A)** Susceptibility, RNA exposure, and anti-MDA5–RNA immune complexes converge on MDA5–interferon signaling. **(B)** Interferon-conditioned immune cells promote epithelial, endothelial, and matrix injury. **(C)** Pulmonary states and outcomes are modified by treatment and infection. ECM, extracellular matrix; IFN, interferon; ILD, interstitial lung disease; MDA5, melanoma differentiation-associated protein 5; RP-ILD, rapidly progressive interstitial lung disease; SPP1, secreted phosphoprotein 1.

### 3.3 Molecular architecture across scales

#### 3.3.1 Susceptibility, RNA sensing, and possible viral triggers

Genetic evidence accords with the defining autoantigen. Population-specific HLA-DRB1 alleles were associated with anti-MDA5+ DM in Japanese and Chinese cohorts (17, 51). A splice-altering WDFY4 variant increased MDA5 signaling and was linked to clinically amyopathic disease; later Chinese data associated WDFY4 variation with RP-ILD (18, 52). The PROMIS registry nominated HLA-DQA1*06:01, and a Japanese multicenter study implicated IFIH1 variants through exploratory exome sequencing and targeted genotyping (53, 54).

These loci place antigen presentation and RNA sensing upstream of the immune response. Cross-ancestry replication and larger event sets should determine whether germline information adds to proximal state measurements when predicting pulmonary course.

Viral exposure is a plausible initiating model. Enteroviral serology associated specific infection histories with anti-MDA5 myositis, and the pandemic-linked MIP-C series combined epidemiological, genetic, and transcriptomic evidence (19, 45); SARS-CoV-2 and broader viral reviews provide context (28, 29). MDA5 sits at the interface of antiviral defense and autoinflammation (55). Anti-MDA5–RNA immune complexes induce interferon-alpha *in vitro*, and patient-derived monoclonal antibodies stimulate interferon-gamma production (56, 57), providing a testable route from transient exposure to sustained immunity.

#### 3.3.2 Interferon activity is the most reproducible molecular signal

Interferon activity is the clearest cross-platform signal. A peripheral-blood type I interferon score was elevated in more than 80% of one cohort, correlated with severity, and identified a poor-survival subgroup, although its cut-off awaits external validation (20). Bulk RNA sequencing showed the strongest type I interferon program in anti-MDA5-positive patients and localized much of it to monocytes (58). Antibody titers and interferon scores generally fell with treatment but followed distinct kinetics (59).

Transcriptomic studies have nominated IFN-beta, EIF2AK2, and IP-10 as markers of pulmonary involvement (60, 61). Serum IFN-lambda3 predicted poor outcome in derivation and validation cohorts, with lung staining implicating macrophages, epithelium, pleura, and intrapulmonary veins (62). Subsequent work described dysregulated type I/III interferon patterns and an IFN-lambda3-dominant high-risk subgroup (63). Multi-tissue transcriptomics identified shared and compartment-specific programs, including DEFA3, RNASE2, and IFI27 as candidate RP-ILD markers (64). Mechanistic studies linked reduced monocyte YTHDF2 to IFNB overexpression and IFN-beta to ZBP1-mediated PANoptosis in T cells (65, 66).

Interferon-stimulated genes are also induced by viral infection, and bulk blood scores depend on cell composition (20, 55, 58). A high score may reflect greater expression per cell, expansion of an interferon-high subset, or both; glucocorticoids and Janus kinase inhibition further alter the signal (59). Serial pretreatment and early-treatment sampling with infection adjudication is therefore required before an interferon assay can guide therapy.

#### 3.3.3 Single-cell data locate a multicellular circuit

Single-cell studies identify the cellular carriers of bulk signals. Ye et al. found expanded antibody-secreting cells, clonally expanded CD8+ memory populations, broad type I interferon activation, and metabolic reprogramming (21). Baseline ISG15+ CD8+ T-cell frequency was associated with one-year survival in 31 patients. The lung analysis, based on one affected patient and public healthy controls, was exploratory.

In eight dermatomyositis patients, including five with anti-MDA5 antibodies, CD14+ monocytes, plasma cells, and an IFI27-high/IFIH1-high monocyte subset were increased (67). Small RP-ILD studies reported expanded CD14+ monocytes, depleted T and natural killer (NK) cells, enhanced antiviral responses, and weaker antigen presentation (68). Paired blood and bronchoalveolar-lavage profiles in five severe cases suggested that transitional circulating monocytes become inflammatory, profibrotic lung macrophages (69). Other studies linked interferon activity to NK-cell loss or dysfunction (70), while patient IgG impaired NK-cell degranulation and enhanced CD16-dependent macrophage activity *in vitro* (71).

These data define an interferon-conditioned circuit spanning monocytes, lung macrophages, plasmablasts, clonally expanded CD8+ T cells, and depleted or dysfunctional NK cells. Associations between immune-cell composition and outcome were extended by a 268-patient flow-cytometric study and a 2026 analysis of CD4+ memory compartments (41, 72). Longitudinal pretreatment cohorts can now test causal ordering and treatment responsiveness.

#### 3.3.4 Proteins connect immune activation with lung injury

Proteomics links immune activation to clinically measurable effector biology. In discovery and validation cohorts, 79 plasma proteins differed between RP-ILD and non-RP-ILD; acute inflammation, complement, and coagulation were enriched, and secreted phosphoprotein 1 (SPP1) had the strongest validated association (22). SPP1 accords with the macrophage-remodeling program in lung single-cell data, although circulating abundance neither identifies its source nor establishes temporal precedence.

A proximity-extension study of 16 Dutch anti-MDA5-positive patients and 15 controls identified broad interferon, IL-1, IL-10, and IL-18 activity in RP-ILD, with differences in IL-6, IL-8, CX3CL1, KRT19, and PD-L1 (73). A larger Olink study across myositis-specific autoantibodies confirmed strong inflammatory-protein perturbation and CX3CL1 in anti-MDA5+ disease, although it addressed classification rather than prospective RP-ILD prediction (74). Galectin-9 also correlates with pulmonary severity (75).

Protein findings resolve into interferon-linked chemokines, myeloid acute-phase or remodeling proteins, and complement–coagulation signals. CXCL10 and galectin-9 are interferon readouts; SPP1 and serum amyloid A1 mark macrophage-rich inflammation; KRT19 and CYFRA21–1 indicate epithelial injury. Because coagulation proteins also change with infection, immobility, and critical illness, modules anchored to cellular source and sampling time are more informative than ranked single proteins.

#### 3.3.5 Metabolism offers a dynamic but easily confounded layer

Metabolomic evidence is early but coherent. Plasma profiling of 25 patients with RP-ILD, 25 with chronic ILD, 24 without ILD, and 41 controls identified broad amino-acid changes and marked tryptophan–kynurenine disturbance in RP-ILD (23). Temporal interpretation is limited by the cross-sectional design and by effects of nutrition, renal function, medication, inflammation, and microbiota.

Integrated plasma and blood-cell profiling identified citrate as a candidate diagnostic marker and dimethylglycine as a correlate of RP-ILD and mortality (76). These findings parallel metabolic reprogramming in single-cell data but lack independent prognostic validation. Fasting, diet, organ failure, hypoxemia, and processing delay must be standardized; flux experiments could distinguish altered pathway activity from passive accumulation.

#### 3.3.6 Priorities for molecular integration

Epigenomic and spatial methods could distinguish transient interferon activity from durable regulation and localize interactions among epithelium, endothelium, macrophages, lymphocytes, and fibroblasts. Existing bronchoalveolar-lavage, biopsy, autopsy, and single-patient datasets provide an initial tissue framework (21, 62, 69).

Most studies measure one molecular layer. A 2026 analysis across broader idiopathic inflammatory myopathy subtypes illustrates coordinated within-patient multi-omics (77), while latent-factor, network-fusion, and multimodal methods offer options for incomplete data and batch structure (32–34). For anti-MDA5 pulmonary stratification, matched blood–lung sampling, harmonized landmarks, and low-dimensional modules are the immediate priorities (**Table 2**).

Cross-platform recurrence is most persuasive when supported by independent cohorts and biological materials, particularly where referral networks or public datasets overlap (20–22, 60, 64). Blood and lung interferon signals may share a driver or represent correlated responses; temporal sampling and perturbation are needed to establish their causal position.

Translation proceeds from discovery through technical confirmation, biological replication, clinical validation at a prespecified landmark, and impact or treatment-interaction testing (16, 78, 79). Stating the completed stage avoids conflating differential features with validated biomarkers.

### 3.4 Candidate endotypes as continuous axes

#### 3.4.1 Interferon and myeloid programs form the dominant core

The interferon–antiviral axis has the broadest evidence, recurring in targeted scores, bulk blood, monocytes, lymphocyte subsets, lung immune cells, chemokines, and type III interferon assays (20, 21, 58, 60, 63–66). Type I interferon may define the syndrome, whereas IFN-lambda3 or the slope of an interferon-stimulated-gene score may refine prognosis (50, 66). A time-varying quantitative axis is therefore more informative than a binary classification (80).

The myeloid axis is closely coupled to interferon but retains distinct cellular and soluble features. Ferritin and soluble CD206 initially indicated macrophage activation; bulk, single-cell, proteomic, and trajectory studies add SPP1, CCL2, IL-18, and monocyte states (22, 50, 60, 61, 67, 68, 81). Longitudinal sampling should test whether this program precedes alveolar injury and distinguish it from infection-related activation.

#### 3.4.2 Adaptive, vascular, and metabolic axes add biological resolution

Adaptive immunity combines activation with cell loss. Plasmablast expansion, B-cell clonality, CD8+ clonal expansion, and autoantibody experiments indicate antigen-driven responses (21, 56, 57), whereas clinical and single-cell studies associate lymphopenia, altered T-cell compartments, and NK-cell depletion with severe disease (39, 67, 68, 70, 72). Expansion may coexist with apoptosis, exhaustion, recruitment, or redistribution; joint measurement of clonality, cell fate, and tissue location would resolve these processes.

Complement, coagulation, and endothelial injury form a plausible vascular axis. Proteomic enrichment, kininogen 1, chemotactic proteins, cutaneous vasculopathy, and diffuse alveolar injury support this link (22, 73, 74). Tissue confirmation and longitudinal sampling can separate local vascular activation from systemic critical illness.

The immunometabolic axis is less mature. Tryptophan–kynurenine disturbance, citrate and dimethylglycine associations, and cellular metabolic reprogramming converge at pathway level (21, 23, 76). Prospective targeted panels should establish timing and incremental information beyond inflammatory severity.

#### 3.4.3 A state space supports dynamic taxonomy

The axes interact: interferon affects monocyte differentiation, lymphocyte survival, and metabolism; macrophages release remodeling and acute-phase proteins; and vascular abnormalities may follow inflammatory lung injury. Patients may therefore be interferon-high, myeloid-high, lymphocyte-depleted, and metabolically perturbed simultaneously, despite HRCT appearances shared with biologically different states (**Figure 3**).

**Figure 3:**
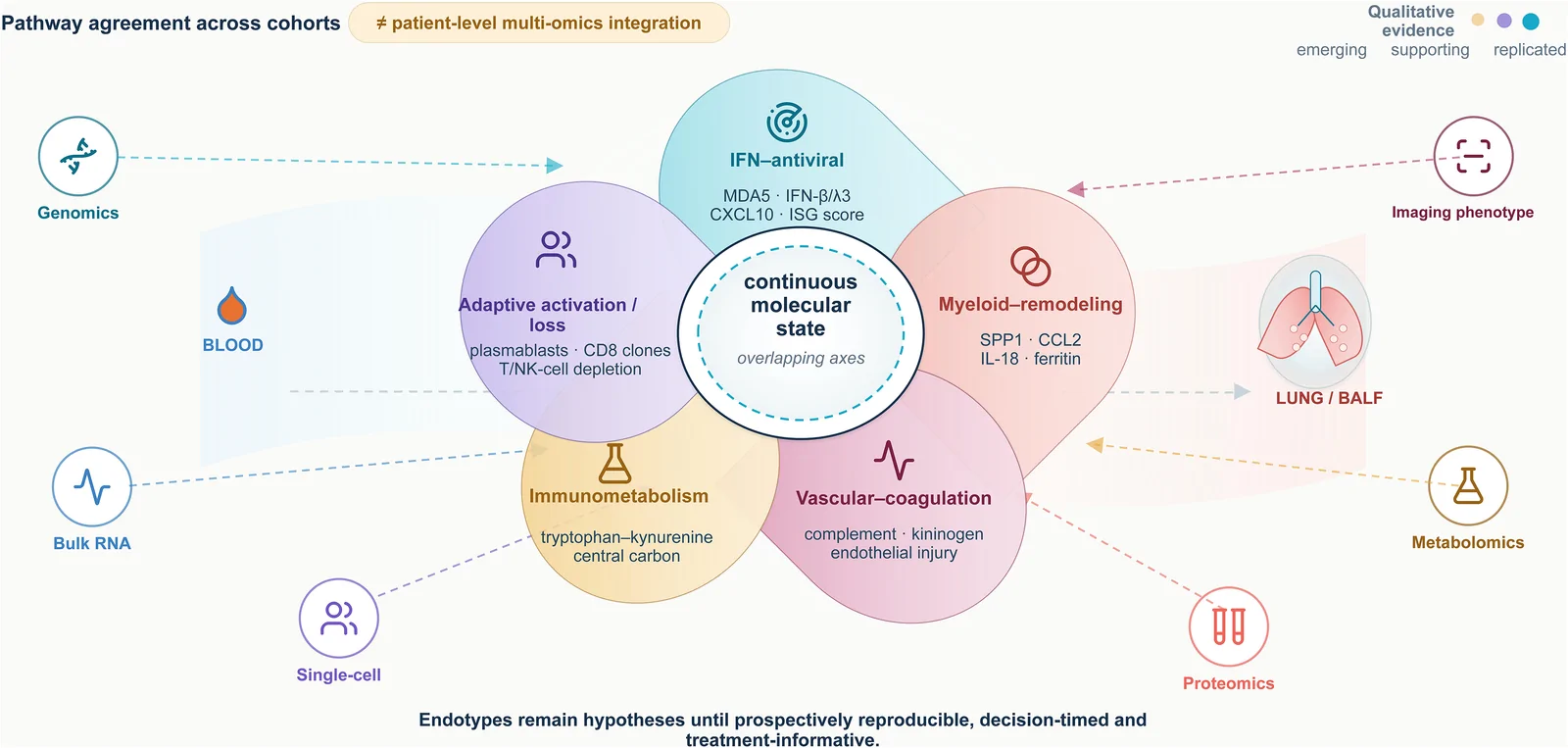
Cross-omics convergence and candidate molecular states in anti-MDA5+ DM-ILD. Evidence converges on interferon–antiviral, myeloid–remodeling, adaptive activation or loss, vascular–coagulation, and immunometabolic axes. Because most layers were studied in separate cohorts, clinical endotypes require prospective, decision-timed validation. BALF, bronchoalveolar-lavage fluid; IFN, interferon; ISG, interferon-stimulated gene; MDA5, melanoma differentiation-associated protein 5; SPP1, secreted phosphoprotein 1.

Continuous axes accommodate mixed biology and shifts after treatment or infection. Discrete classes matter only when a reproducible rule assigns new patients. An actionable endotype should replicate across cohorts and ancestries, withstand sampling and analytical variation, show defined temporal behavior, improve prediction beyond stage, infection, and treatment, and identify differential benefit from a specified intervention. The interferon axis currently best meets the first three criteria (16); **Table 3** summarizes the evidence and validation tests.

### 3.5 What current prediction models estimate

#### 3.5.1 Clinical clusters provide a foundation for mechanistic validation

Unsupervised analyses repeatedly group rash, arthritis, lymphocyte count, immune-cell composition, inflammatory markers, and lung involvement into clinically recognizable patterns (8, 24, 39–44). Because each solution depends on feature selection and analytical choices, independent molecular and longitudinal evidence is needed before assigning mechanistic meaning.

Endotype discovery should test stability across resampling, preprocessing alternatives, and an external cohort. Immune-cell clusters become mechanistically anchored when composition maps to stable biology and a prospectively tested treatment interaction (42, 43). A consensus rule can then assign new patients without reclustering, while multiview methods preserve layer-specific structure when the number of independent patients is adequate (32, 77).

#### 3.5.2 Clinical models provide an established baseline

Clinical scores provide accessible baselines. CROSS combines CRP, anti-Ro52 positivity, disease duration of three months or less, and male sex; development in 170 patients and geographic validation in 85 yielded time-dependent AUCs above 0.80 and very-high-risk 1-year RP-ILD incidences of 71.3% and 85.7% (25). CRAFT combines the CRP-to-albumin ratio, RDW-CV, fever, and CD3+ T-cell count; bootstrap validation in 53 patients with 21 events yielded an AUC of 0.93 (82). FLAW uses fever, LDH, age, and white-cell count; a small internal subset reproduced ordered RP-ILD risk (83). These models provide practical calibration and validation baselines.

Mortality models address different populations and landmarks. FLAIR combines ferritin, LDH, antibody grade, HRCT score, and RP-ILD in amyopathic DM-ILD (84). The MCK model, developed in 497 patients and validated in 111, combines anti-MDA5 status, CRP, and KL-6 in polymyositis/dermatomyositis-ILD (85). Anti-MDA5-specific models include oxygenation, lymphocyte subsets, CYFRA21-1, anti-Ro52, fever, ferritin, carcinoembryonic antigen, pleural effusion, and CT severity (86–88). Their performance must be interpreted against the target population, horizon, landmark, and validation design.

A six-hospital Chinese study of 509 patients predicted three-month mortality from RP-ILD, erythrocyte sedimentation rate, albumin, age, CRP, aspartate aminotransferase, LDH, and neutrophil-to-lymphocyte ratio (26). Logistic regression outperformed five flexible algorithms, with an internal AUC of 0.866 and external AUCs of 0.836 and 0.915; calibration, decision curves, SHapley Additive exPlanations, and a web tool were reported. Strong tabular predictors may favor a transparent model over greater algorithmic complexity.

Models containing established RP-ILD estimate mortality after progression; prevention models require an earlier landmark. Retrospective models also reflect source-cohort treatment, so recalibration across treatment eras matters. FLATCAN, derived from 621 patients, used a conventional Cox model that outperformed five alternatives and achieved a C-index of 0.815 with strong time-dependent AUCs (89). Internal and temporal validation support subsequent geographic evaluation.

#### 3.5.3 Imaging adds spatial information and benefits from domain harmonization

HRCT contains information beyond visual extent. Xu et al. modeled 6-month mortality in 228 patients; C-indices were 0.88 in training and testing, 0.83 in temporal validation, and 0.84 externally (90). Li et al. combined disease duration, dyspnea, and three radiomic features in 103 patients to predict RP-ILD (test AUC, 0.812) (91). He et al. combined bilateral-lung radiomics, consolidation score, LDH, and infection in 119 training, 41 internal-validation, and 29 external-validation patients; nomogram AUCs were 0.882 and 0.916 in the validation cohorts (27). Semiquantitative HRCT plus clinical data yielded an internally validated AUC of 0.842 in 67 patients (92).

Radiomic transportability depends on harmonized acquisition, reconstruction, slice thickness, segmentation, feature definition, preprocessing, and infection adjudication. Compliance with the Image Biomarker Standardization Initiative and CLAIM, together with scanner-stratified external validation, supports reproducibility (93, 94). Deep-learning models require the same domain-shift testing.

#### 3.5.4 Performance must be read in the context of intended use

Time zero is often ambiguous. Short disease duration may reflect early hazard or referral delay (25); cohorts begin at antibody confirmation, admission, or ILD diagnosis (26, 82, 84, 86), and post-deterioration predictors may leak outcome information. The intended action—monitoring, admission, escalation, or referral—must therefore be defined before modeling.

Outcome and horizon must match the decision. Three-month death combines autoimmune injury, infection, treatment toxicity, and other causes (26, 46); RP-ILD varies by interval and criteria (10, 14); and transplantation-free survival depends partly on access (49). Competing-risk or multistate analyses are preferable when infection, transplantation, or non-pulmonary death changes interpretation.

Transportability assessment should compare case mix, measurement, treatment era, predictor distributions, missingness, and outcome incidence. Recalibration can correct shifted baseline risk; altered predictor–outcome relations require new validation. A single external AUC cannot distinguish these mechanisms.

Clinical utility requires absolute risk as well as discrimination. Calibration-in-the-large, calibration slope, and plots should accompany AUC; Brier score and decision-curve analysis add overall accuracy and threshold-specific net benefit (95). Sample size should reflect events, predictor parameters, anticipated fit, and precision (96), with efficient resampling followed by independent validation.

Robust development addresses retrospective sampling, few events, complete-case analysis, univariable screening, dichotomization, and feature selection outside resampling. Calibration uncertainty and validation beyond the source region should be reported. TRIPOD+AI and PROBAST+AI structure reporting of participants, outcome, time zero, missing data, model specification, and validation (78, 79).

SHAP values and nomograms describe how a model uses inputs; they do not establish mechanism or treatment benefit. Clinical interpretation requires absolute risk and uncertainty, mechanistic claims require molecular evidence, and treatment selection requires effect modification. **Table 1** compares representative prediction and monitoring studies by population, landmark, outcome, horizon, and validation design. Across the studies summarized in **Table 1**, methodological limitations commonly included retrospective designs, limited event numbers, incomplete calibration reporting, and limited external validation. Performance estimates should therefore be interpreted in relation to the study population, prediction target, landmark, and validation design.

### 3.6 Connecting molecular state to dynamic risk

#### 3.6.1 Routine biomarkers are compressed views of upstream biology

Routine predictors are compressed views of biology. CRP, ferritin, and neutrophil-to-lymphocyte ratio reflect systemic inflammation; lymphocyte and CD3+ T-cell counts reflect depletion, redistribution, and treatment. Anti-Ro52 is associated with RP-ILD and mortality (97, 98). KL-6 and CYFRA21–1 index epithelial injury (99, 100), while IL-15 and BAFF reflect immune activation (101, 102). Anti-MDA5 titer and IgG subclass composition capture humoral activity (103–105). LDH, oxygenation, physiology, and HRCT record downstream injury (12–15). Several variables may therefore project the same underlying process.

These mappings are many-to-many: ferritin rises in infection, LDH is not lung-specific, glucocorticoids alter lymphocytes, and HRCT ground-glass opacity has several causes. A molecular assay is valuable when it resolves decision-relevant ambiguity. Incremental value should be tested against a credible clinical–imaging model using calibration and net benefit at prespecified thresholds.

#### 3.6.2 Baseline risk provides the model backbone

A staged model begins with a parsimonious, calibrated, externally validated clinical–imaging backbone (95, 96), then tests one compact, nonredundant module: an interferon score, SPP1-centered myeloid panel, lymphocyte-state assay, targeted metabolite panel, or reproducible imaging score. Module-wise evaluation shows whether additional testing changes risk where management is uncertain.

Routine measurements can serve all patients, with molecular testing concentrated in an intermediate-risk range where it may change management. Decision analysis should define that range; established respiratory failure already demands urgent care.

At presentation, models should distinguish no pulmonary involvement, ILD without documented acceleration, and RP-ILD already in progress. Pretreatment blood modules should be analytically validated, with HRCT and physiology as the backbone. Outputs should report absolute risk over a stated horizon and uncertainty when data are missing or outside the validated range.

A second landmark—days 3–7 in hospital or a prespecified early outpatient review—can incorporate oxygenation, laboratory and molecular slopes, clinically indicated imaging, treatment, and infection. Discordance may be informative: persistently high CRP with a falling interferon module differs from parallel rises in interferon, myeloid proteins, and CT extent. Such patterns require prospective testing but illustrate decision-timed updating.

Trajectories may outperform baseline thresholds during the early hazard. Ferritin trajectories separated mortality risk (106), and failure of IFN-lambda3 to fall by one month was associated with death or relapse in 24 patients (80). Among 310 six-month survivors, faster anti-MDA5 seroconversion predicted better long-term control; a reduction of at least 33% by three months identified this trajectory with an AUC of 0.86 (50). The last result applies to survivors beyond the earliest high-risk period.

Landmark models update risk at fixed decisions; joint longitudinal–survival models link trajectories to events; and multistate models represent transitions among no ILD, stable ILD, RP-ILD, recovery, relapse, and death. Relevant landmarks include first specialist assessment, treatment initiation, days 3–7, and early outpatient review (**Figure 4**).

**Figure 4:**
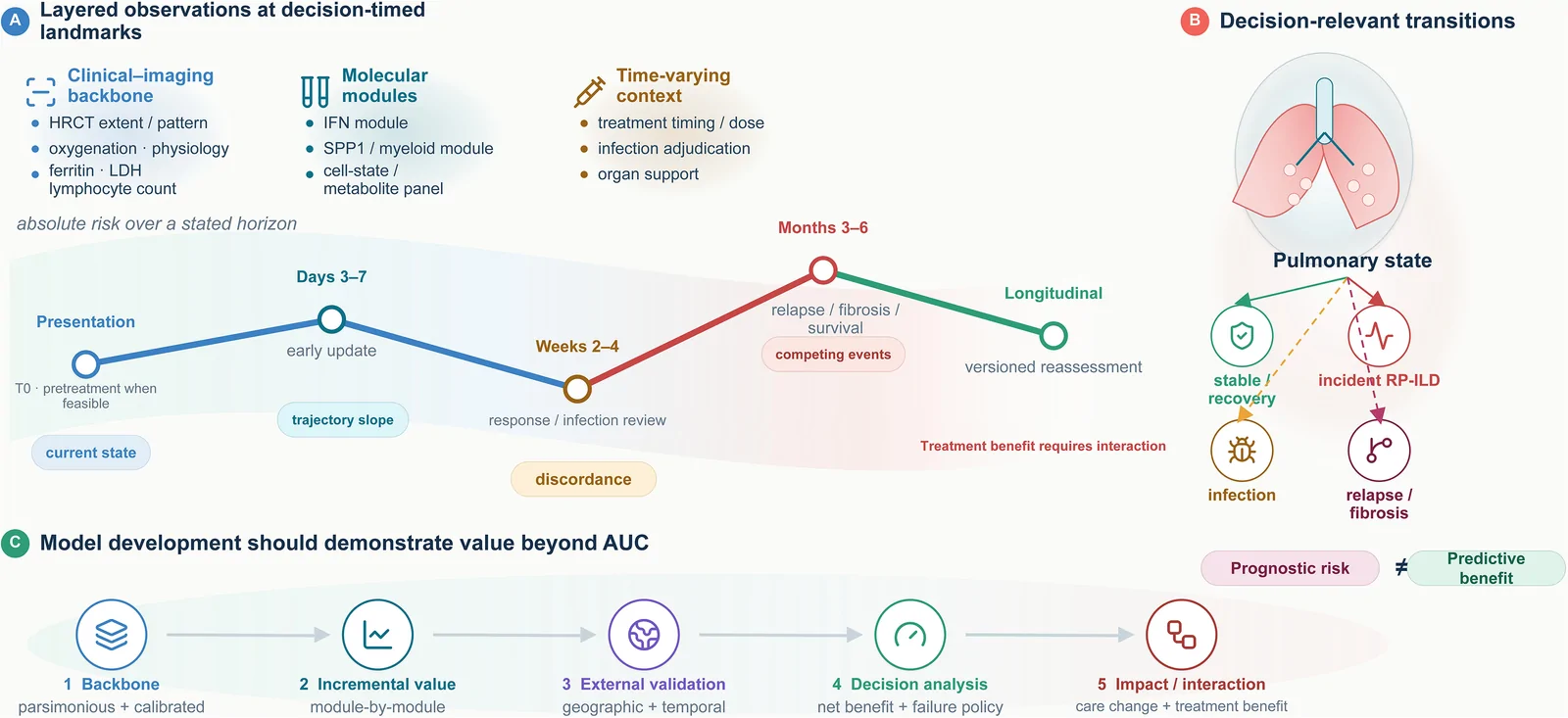
Decision-timed framework for dynamic risk prediction in anti-MDA5+ DM-ILD. **(A)** A clinical–imaging backbone is updated at prespecified landmarks with molecular modules and time-varying context. **(B)** Models estimate state transitions while separating prognosis from treatment-benefit prediction. **(C)** Evaluation includes calibration, incremental value, external validation, decision analysis, and prospective impact. AUC, area under the receiver-operating-characteristic curve; HRCT, high-resolution computed tomography; IFN, interferon; LDH, lactate dehydrogenase; RP-ILD, rapidly progressive interstitial lung disease; SPP1, secreted phosphoprotein 1.

#### 3.6.3 Treatment response is a different prediction problem

Once treatment begins, biomarker meaning becomes regimen-dependent, sampling intensifies during deterioration, and death truncates trajectories. Longitudinal models must therefore include treatment timing and dose, infection, and organ support as time-varying exposures.

Prognostic and predictive biomarkers must be distinguished (16). High interferon activity may indicate poor outcome under any treatment or greater benefit from Janus kinase inhibition. Upfront glucocorticoid, tacrolimus, and cyclophosphamide improved survival prospectively in Japan, whereas a later multicenter cohort found no uniform triple-therapy benefit (47, 48). Tofacitinib, rituximab, and plasma exchange have cohort, case-series, or meta-analytic support (107–110). In 515 patients across five centers, inverse-probability weighting associated tofacitinib with better one-year lung-transplantation-free survival than calcineurin inhibitors, although the comparison remained nonrandomized (49).

Biomarker-stratified trials can test endotype-directed care. A platform design could apply a common clinical-risk definition, measure a compact pretreatment panel, and randomize within biological strata. When trials are infeasible, target-trial emulation should prespecify eligibility, treatment strategies, assignment, time zero, follow-up, outcome, and causal contrast (111). A reproducible interaction with adequate precision is required to claim treatment-benefit prediction.

## 4 Discussion

### 4.1 Interpretation and research priorities

Evidence converges on interferon-conditioned immunity, myeloid activation, adaptive-cell perturbation, and lung-injury proteins, while multicenter models provide strong clinical baselines. The next step is integration: pretreatment sampling and external assignment rules can refine immune-cell states (42, 43), and linking molecular modules to prediction cohorts can test incremental value and treatment interaction.

Prospective inception cohorts should enroll at first suspicion or antibody confirmation and collect pretreatment samples when feasible. Core data include symptoms, oxygenation, physiology, quantitative antibodies, routine tests, HRCT metadata, treatment, and infection. Sampling at days 3–7, weeks 2–4, months 3 and 6, and clinical events would distinguish incident RP-ILD, established progression, infection, early death, relapse, and fibrosis across regions.

Decision-timed assays require standardized processing, storage, fasting, organ function, and infection status. Single-cell studies need batch-balanced processing and patient-level inference; proteomic and metabolomic studies need pooled controls, drift correction, and blinded replication. HRCT pipelines should retain acquisition metadata and lock segmentation and features before validation (93, 94).

Integration must respect effective sample size. Feature concatenation amplifies batch effects; latent-factor and multiview methods may be unstable in small cohorts, whereas late integration tolerates missing modalities but loses interactions (32–34). Biology-informed modules and sparse hierarchical models are preferable to unrestricted deep networks, with preprocessing and tuning confined to resampling.

Model development requires a prespecified estimand, time zero, and intended action. Sample size should reflect events, parameters, anticipated fit, and precision (96), followed by resampling and locked geographic and temporal validation. TRIPOD+AI and PROBAST+AI support reporting of discrimination, calibration, Brier score, net benefit, uncertainty, missing-assay policies, model versions, and subgroup performance (78, 79).

Longitudinal analyses should account for deterioration-driven testing and treatment. Joint models address informative measurement and event processes, inverse-probability methods reduce measured treatment-selection bias, and multistate models represent pulmonary transitions. The decisive test is whether state-guided treatment improves outcomes.

The framework yields explicit advancement criteria. A molecular module moves toward practice when it improves calibration or net benefit beyond a clinical–imaging model; a biological axis gains endotype status through stability across treatment, infection, and assay conditions; and a prognostic marker becomes treatment-informative through a reproducible interaction. A compact assay may still matter if it changes decisions within a prespecified intermediate-risk group. The five molecular programs synthesized here are better conceptualized as overlapping continuous biological axes than as fixed discrete endotypes. Together, they provide a biologically grounded framework for prospective endotype validation and treatment-aware stratification, with the interferon axis currently showing the strongest cross-platform reproducibility.

### 4.2 Limitations

Several limitations should be acknowledged. Research on molecular endotyping and dynamic risk prediction in anti-MDA5+ DM-ILD is evolving rapidly, and the current evidence map will require continued refinement as new multi-omics and prediction studies emerge. Differences in the molecular layers examined, prediction endpoints, and reporting practices also limit direct comparison across studies, while head-to-head evaluation of candidate endotypes remains scarce. As this review was designed to systematically map and integrate the available evidence rather than to generate pooled effect estimates, the proposed framework will require further validation in prospective multicenter studies. The evidence map may also be influenced by the restriction to English-language publications.

## 5 Conclusion

Anti-MDA5+ DM-ILD is serologically coherent but biologically multidimensional. The strongest evidence supports an interferon–antiviral program coupled to monocyte–macrophage inflammation and remodeling; adaptive-cell perturbation, vascular injury, and altered metabolism add complementary axes. Immune-cell clusters provide starting points for endotype validation. Clinical and imaging models capture substantial early risk, and explicit landmarks plus parsimonious molecular modules may improve utility and transportability.

A staged longitudinal strategy can convert cross-omics convergence into decision-specific prediction: validate a clinical–imaging backbone, add nonredundant molecular modules, and update risk while accounting for treatment and infection. Clinical credibility ultimately requires reproducible measurement, transportability, and demonstrable improvement in treatment decisions or patient outcomes.

## Data Availability

The original contributions presented in the study are included in the article/**Supplementary Material**. Further inquiries can be directed to the corresponding author.
