# Supplementary material for: Candidate molecular endotypes and dynamic risk prediction in anti-MDA5-positive dermatomyositis-associated interstitial lung disease: a systematic scoping review
Source: Front Immunol. 2026 Oct 9;17:1979138. doi: 10.3389/fimmu.2026.1979138 (PMC13690796; doi:10.3389/fimmu.2026.1979138)
Supplement: Supplementary file 1 [file SupplementaryFile1.docx]

# **Supplementary Methods 1. Complete database-specific search strategies**

**Database-specific literature search strategies**

**Search date:** 22 August 2026

**Coverage:** Database inception through 22 August 2026; no lower date limit.

Information sources and platforms: PubMed, Web of Science, Ovid and Embase

Searches combined the anti-MDA5/IFIH1 axis, dermatomyositis, and interstitial lung disease, including rapidly progressive interstitial lung disease. No language or species filters were applied at the database-search stage; non-English and non-human records were excluded during subsequent screening. Search strings are reported in the form used on each platform.

Records retrieved before deduplication: PubMed, 877; Web of Science, 814; Ovid , 612; Embase, 1,863; total, 4,166.

## **PubMed**

```
#1  IFIH1[tiab] OR "MDA5"[tiab] OR "anti-MDA5"[tiab] OR "anti MDA5"[tiab]
     OR "MDA5 antibod*"[tiab]
     OR "anti-melanoma differentiation-associated gene 5"[tiab]
     OR "anti-melanoma differentiation associated protein 5"[tiab]
     OR "MDA5-positive"[tiab] OR "MDA5 positive"[tiab]
#2  dermatomyositis[tiab] OR dermatomyositides[tiab] OR "Dermatomyositis"[Mesh]
#3  "Lung Diseases, Interstitial"[Mesh] OR "Pulmonary Fibrosis"[Mesh]
     OR "interstitial lung disease*"[tiab] OR ILD[tiab]
     OR "pulmonary fibrosis"[tiab] OR "rapidly progressive"[tiab] OR "RP-ILD"[tiab]
#4  #1 AND #2 AND #3

```

## **Web of Science**

```
#1  TS=("anti-MDA5" OR "anti MDA5" OR "MDA5 antibod*" OR
     "anti-melanoma differentiation-associated gene 5" OR
     "anti-melanoma differentiation associated protein 5" OR
     "MDA5-positive" OR "MDA5 positive" OR IFIH1)
#2  TS=(dermatomyositis OR dermatomyositides)
#3  TS=("interstitial lung disease*" OR ILD OR "pulmonary fibrosis" OR
     "rapidly progressive" OR RP-ILD)
#4  #1 AND #2 AND #3
```

## Ovid

((exp Dermatomyositis/ OR dermatomyosit$.mp.) AND
(exp Lung Diseases, Interstitial/ OR exp Pulmonary Fibrosis/ OR
interstitial lung disease$.mp. OR ILD.mp. OR pulmonary fibrosis.mp. OR
rapidly progressive.mp. OR RP-ILD.mp.) AND
(exp Interferon-Induced Helicase, IFIH1/ OR IFIH1.mp. OR MDA5.mp. OR
anti-MDA5.mp. OR "anti MDA5".mp. OR MDA5 antibod$.mp. OR
"anti-melanoma differentiation-associated gene 5".mp. OR
"anti-melanoma differentiation associated protein 5".mp. OR
MDA5-positive.mp. OR "MDA5 positive".mp.))

## **Embase**

('dermatomyositis'/exp OR 'dermatomyositis':ti,ab,kw OR dermatomyosit*:ti,ab,kw)
AND ('interstitial lung disease'/exp OR 'lung fibrosis'/exp OR
'interstitial lung disease':ti,ab,kw OR 'ild':ti,ab,kw OR 'lung fibrosis':ti,ab,kw OR
'pulmonary fibrosis':ti,ab,kw OR 'rapidly progressive':ti,ab,kw OR 'rp-ild':ti,ab,kw)
AND ('melanoma differentiation associated protein 5'/exp OR
'interferon induced helicase IFIH1'/exp OR 'ifih1':ti,ab,kw OR 'mda5':ti,ab,kw OR
'anti-mda5':ti,ab,kw OR 'anti mda5':ti,ab,kw OR 'mda5 antibod*':ti,ab,kw OR
'anti-melanoma differentiation-associated gene 5':ti,ab,kw OR
'anti-melanoma differentiation associated protein 5':ti,ab,kw OR
'mda5-positive':ti,ab,kw OR 'mda5 positive':ti,ab,kw)
