# Supplementary material for: Candidate molecular endotypes and dynamic risk prediction in anti-MDA5-positive dermatomyositis-associated interstitial lung disease: a systematic scoping review
Source: Front Immunol. 2026 Oct 9;17:1979138. doi: 10.3389/fimmu.2026.1979138 (PMC13690796; doi:10.3389/fimmu.2026.1979138)
Supplement: Supplementary file 2 [file SupplementaryFile2.docx]

**Supplementary Table S1. Characteristics and contributions of included evidence sources**

This table reconciles the 95 sources in the PRISMA-ScR flow diagram with the numbered reference list. Eighty primary reports provide source-specific clinical, molecular, biomarker, prediction, imaging, or treatment evidence; 15 secondary syntheses provide context and were not counted as independent replication of primary findings.

*Design and reporting features were charted descriptively rather than converted into formal risk-of-bias ratings. Detailed sample sizes and performance estimates for the principal molecular and prediction studies appear in Tables 1 and 3 of the main manuscript. Funding of individual evidence sources was not systematically charted or used for eligibility or weighting; review-level funding is reported in the main manuscript. Sixteen methodological or contextual references (16, 32–37, 43, 55, 78, 79, 93–96, and 111) were not counted among the 95 evidence sources.*

**Table S1A. Primary evidence sources (n=80)**

| **Ref.** | **Evidence source** | **Design / population or material** | **Index / data type** | **Pulmonary or clinical endpoint** | **Principal finding or contribution** | **Appraisal note** |
| --- | --- | --- | --- | --- | --- | --- |
| 4 | Sato S et al. (2005). Autoantibodies to a 140-kd polypeptide, CADM-140, in Japanese patients with clinically amyopathic dermatomyositis. | Clinical phenotype / outcome cohort; Clinically amyopathic DM / DM-ILD | Clinical phenotype and outcome characterization | clinical or molecular phenotype | First described the CADM-140 autoantibody in clinically amyopathic dermatomyositis. | Observational evidence interpreted in relation to setting, landmark, treatment era, and outcome definition. |
| 5 | Sato S et al. (2009). RNA helicase encoded by melanoma differentiation-associated gene 5 is a major autoantigen in patients with clinically amyopathic dermatomyositis: Association with rapidly progressive interstitial lung disease. | Clinical phenotype / outcome cohort; Clinically amyopathic DM / DM-ILD | Clinical phenotype and outcome characterization | RP-ILD; ILD phenotype/severity | Identified MDA5 as the CADM-140 autoantigen and linked anti-MDA5 positivity to RP-ILD. | Observational evidence interpreted in relation to setting, landmark, treatment era, and outcome definition. |
| 8 | Allenbach Y et al. (2020). Different phenotypes in dermatomyositis associated with anti-MDA5 antibody: Study of 121 cases. | Prediction / stratification study; Anti-MDA5+ DM or DM-ILD | Clinical or immunophenotypic clustering | clinical or molecular phenotype | Identified three clinically distinct anti-MDA5+ DM phenotypes dominated by pulmonary, rheumatic, or vasculopathic features. | Observational evidence interpreted in relation to setting, landmark, treatment era, and outcome definition. |
| 9 | Faria MSMS et al. (2025). Different phenotypic manifestations between Brazilian and Japanese anti-MDA5 antibody-positive dermatomyositis: an international tricentric longitudinal study. | Clinical phenotype / outcome cohort; Brazilian and Japanese anti-MDA5+ DM cohorts | Clinical phenotype and outcome characterization | clinical or molecular phenotype | Demonstrated marked Brazilian-Japanese differences in pulmonary, muscle, cutaneous, and articular expression. | Observational evidence interpreted in relation to setting, landmark, treatment era, and outcome definition. |
| 10 | Chen X et al. (2023). Clinical, radiological and pathological features of anti-MDA5 antibody-associated interstitial lung disease. | Clinical phenotype / outcome cohort; Anti-MDA5+ DM or DM-ILD | Clinical phenotype and outcome characterization | ILD phenotype/severity | Characterized radiological and pathological ILD patterns and their relation to rapid progression. | Observational evidence interpreted in relation to setting, landmark, treatment era, and outcome definition. |
| 17 | Gono T et al. (2012). Brief report: Association of HLA-DRB1*01:01/*04:05 with susceptibility to anti-melanoma differentiation-associated gene 5 antibody-positive dermatomyositis in the Japanese population. | Molecular / patient-derived mechanistic study; Japanese anti-MDA5+ DM cohort; germline HLA genotyping | Germline genetic association / sequencing | clinical or molecular phenotype | Associated population-specific HLA-DRB1 alleles with susceptibility to anti-MDA5+ DM. | Association or discovery evidence; assay standardization, sampling landmark, and independent replication were charted. |
| 18 | Kochi Y et al. (2018). Splicing variant of WDFY4 augments MDA5 signalling and the risk of clinically amyopathic dermatomyositis. | Molecular / patient-derived mechanistic study; CADM n=576; germline association, eQTL, and functional signaling | Germline genetic association / sequencing | clinical or molecular phenotype | Linked a WDFY4 splice variant to clinically amyopathic DM and showed augmented MDA5 signaling. | Association or discovery evidence; assay standardization, sampling landmark, and independent replication were charted. |
| 19 | Jayaraman S et al. (2025). Comprehensive Enteroviral Serology Links Infection and Anti-Melanoma Differentiation-Associated Protein 5 Dermatomyositis. | Clinical phenotype / outcome cohort; Anti-MDA5+ DM or DM-ILD | Viral serology, infection, or pandemic context | clinical or molecular phenotype | Linked enteroviral serological histories with anti-MDA5 dermatomyositis. | Observational evidence interpreted in relation to setting, landmark, treatment era, and outcome definition. |
| 20 | Qian J et al. (2023). Type I interferon score is associated with the severity and poor prognosis in anti-MDA5 antibody-positive dermatomyositis patients. | Molecular / patient-derived mechanistic study; Autoimmune disease n=262 (anti-MDA5+ DM n=61) plus 58 controls; baseline and serial PBMC assays | Interferon-pathway measurement | mortality/prognosis | Showed that a high peripheral type I interferon score tracked disease severity and poorer short-term survival. | Association or discovery evidence; assay standardization, sampling landmark, and independent replication were charted. |
| 21 | Ye Y et al. (2022). Single-cell profiling reveals distinct adaptive immune hallmarks in MDA5+ dermatomyositis with therapeutic implications. | Molecular / patient-derived mechanistic study; Active anti-MDA5+ DM n=7, paired remission n=3, disease controls n=5, controls n=4; survival cohort n=31 | Single-cell transcriptomics and immune-cell states | clinical or molecular phenotype | Resolved plasmablast, clonal CD8+ T-cell, interferon, and metabolic states at single-cell resolution. | Mechanistic discovery evidence; sample size, treatment timing, or temporal ordering informs interpretation. |
| 22 | Qiu Y et al. (2024). Proteomic profiling identifies SPP1 associated with rapidly progressive interstitial lung disease in anti-MDA5-positive dermatomyositis. | Molecular / patient-derived mechanistic study; Discovery: anti-MDA5+ DM n=20 and controls n=10; validation: RP-ILD n=20, non-RP-ILD n=20, controls n=20 | Serum/plasma protein profiling | RP-ILD; ILD phenotype/severity | Identified and independently validated an SPP1-centered plasma-protein signature associated with RP-ILD. | Association or discovery evidence; assay standardization, sampling landmark, and independent replication were charted. |
| 23 | Liu H et al. (2026). Tryptophan metabolism alteration is associated with rapidly progressive interstitial lung disease of anti-MDA5+ dermatomyositis. | Molecular / patient-derived mechanistic study; RP-ILD n=25, chronic ILD n=25, no ILD n=24, and controls n=41 | Metabolomic or metabolic-pathway profiling | RP-ILD; ILD phenotype/severity | Identified tryptophan-kynurenine and broader amino-acid disturbances associated with RP-ILD. | Association or discovery evidence; assay standardization, sampling landmark, and independent replication were charted. |
| 24 | Xu L et al. (2023). Identification of Three Different Phenotypes in Anti-Melanoma Differentiation-Associated Gene 5 Antibody-Positive Dermatomyositis Patients: Implications for Prediction of Rapidly Progressive Interstitial Lung Disease. | Prediction / stratification study; Anti-MDA5+ DM or DM-ILD | Clinical prediction or risk-stratification model | RP-ILD; ILD phenotype/severity | Derived three clinical phenotypes with different RP-ILD risks. | Observational evidence interpreted in relation to setting, landmark, treatment era, and outcome definition. |
| 25 | Wang L et al. (2024). Rapidly progressive interstitial lung disease risk prediction in anti-MDA5 positive dermatomyositis: the CROSS model. | Prediction / stratification study; Anti-MDA5+ DM n=170 development and n=85 geographic validation | Clinical prediction or risk-stratification model | Incident RP-ILD within 1 year | Developed and geographically validated the CROSS score for incident RP-ILD. | Independent, geographic, or temporal validation reported; population and landmark remain relevant to transportability. |
| 26 | Li H et al. (2025). Mortality Risk Prediction in Patients With Antimelanoma Differentiation-Associated, Gene 5 Antibody-Positive, Dermatomyositis-Associated Interstitial Lung Disease: Algorithm Development and Validation. | Prediction / stratification study; Anti-MDA5+ DM or DM-ILD | Clinical prediction or risk-stratification model | mortality/prognosis; ILD phenotype/severity | Developed and externally tested a routine-clinical-variable model for 3-month mortality. | Independent, geographic, or temporal validation reported; population and landmark remain relevant to transportability. |
| 27 | He W et al. (2024). Radiomics based on HRCT can predict RP-ILD and mortality in anti-MDA5 + dermatomyositis patients: a multi-center retrospective study. | Prediction / stratification study; Anti-MDA5+ DM-ILD n=119 training, n=41 internal validation, and n=29 external validation | HRCT phenotype, score, or radiomics | Incident RP-ILD; subsequent mortality association | Externally validated an HRCT-radiomic nomogram for RP-ILD and linked the score to mortality. | Independent, geographic, or temporal validation reported; population and landmark remain relevant to transportability. |
| 38 | Tiniakou E et al. (2023). Anti-MDA5-positive dermatomyositis and remission in a single referral centre population. | Clinical phenotype / outcome cohort; Anti-MDA5+ DM or DM-ILD | Clinical phenotype and outcome characterization | clinical or molecular phenotype | Documented a subset achieving sustained drug-free remission in a North American referral cohort. | Observational evidence interpreted in relation to setting, landmark, treatment era, and outcome definition. |
| 39 | Jin Q et al. (2023). Peripheral lymphocyte count defines the clinical phenotypes and prognosis in patients with anti-MDA5-positive dermatomyositis. | Prediction / stratification study; Anti-MDA5+ DM or DM-ILD | Clinical or immunophenotypic clustering | mortality/prognosis | Used lymphocyte count to define clinically and prognostically distinct anti-MDA5+ DM phenotypes. | Observational evidence interpreted in relation to setting, landmark, treatment era, and outcome definition. |
| 40 | Yang Q et al. (2022). Anti-melanoma differentiation-associated 5 gene antibody-positive dermatomyositis exhibit three clinical phenotypes with different prognoses. | Prediction / stratification study; Anti-MDA5+ DM or DM-ILD | Clinical or immunophenotypic clustering | clinical or molecular phenotype | Reported three clinical phenotypes with different pulmonary and survival profiles. | Observational evidence interpreted in relation to setting, landmark, treatment era, and outcome definition. |
| 41 | Zhang Y et al. (2026). Peripheral blood immunophenotypic diversity in patients with anti-MDA5+ dermatomyositis and its impact on prognosis. | Clinical phenotype / outcome cohort; Anti-MDA5+ DM flow-cytometry cohort; n=268 | Clinical or immunophenotypic clustering | mortality/prognosis | Linked peripheral immunophenotypic diversity to prognosis in a large flow-cytometry cohort. | Observational evidence interpreted in relation to setting, landmark, treatment era, and outcome definition. |
| 42 | Guo R et al. (2024). Disease-associated immune cell endotypes in anti-MDA5-positive dermatomyositis using unbiased hierarchical clustering. | Prediction / stratification study; Anti-MDA5+ DM inpatients n=123; activation-marker subset n=33 | Clinical or immunophenotypic clustering | clinical or molecular phenotype | Identified admission immune-cell endotypes associated with RP-ILD, infection, treatment intensity, and early mortality. | Observational evidence interpreted in relation to setting, landmark, treatment era, and outcome definition. |
| 44 | He W et al. (2026). Risk stratification in anti-MDA5+ dermatomyositis-related interstitial lung disease by using cluster analysis. | Prediction / stratification study; Anti-MDA5+ DM or DM-ILD | Clinical prediction or risk-stratification model | ILD phenotype/severity | Derived cluster-based risk strata for anti-MDA5+ DM-ILD. | Observational evidence interpreted in relation to setting, landmark, treatment era, and outcome definition. |
| 45 | David P et al. (2024). MDA5-autoimmunity and interstitial pneumonitis contemporaneous with the COVID-19 pandemic (MIP-C). | Clinical phenotype / outcome cohort; Human DM/IIM cohorts or patient-derived material | Viral serology, infection, or pandemic context | ILD phenotype/severity | Connected pandemic-period MDA5 autoimmunity and pneumonitis with epidemiological, genetic, and transcriptomic evidence. | Observational evidence interpreted in relation to setting, landmark, treatment era, and outcome definition. |
| 46 | Gao C et al. (2024). Pneumocystis jirovecii pneumonia increases the 3-months mortality of anti-MDA5-antibody-positive dermatomyositis patients. | Clinical phenotype / outcome cohort; Anti-MDA5+ DM cohort n=92; opportunistic-infection and 3-month outcome analysis | Clinical phenotype and outcome characterization | mortality/prognosis | Associated Pneumocystis jirovecii pneumonia with higher 3-month mortality. | Observational evidence interpreted in relation to setting, landmark, treatment era, and outcome definition. |
| 47 | Tsuji H et al. (2020). Multicenter Prospective Study of the Efficacy and Safety of Combined Immunosuppressive Therapy With High-Dose Glucocorticoid, Tacrolimus, and Cyclophosphamide in Interstitial Lung Diseases Accompanied by Anti-Melanoma Differentiation-Associated Gene 5-Positive Dermatomyositis. | Therapeutic cohort / case series; Anti-MDA5+ DM or DM-ILD | Treatment exposure and clinical outcome | ILD phenotype/severity; treatment response/control | Prospectively evaluated upfront glucocorticoid-tacrolimus-cyclophosphamide combination therapy. | Treatment-context evidence; selection and time-varying treatment were considered when interpreting outcomes. |
| 48 | You H et al. (2025). Triple-combination therapy did not improve prognosis in anti-MDA5 positive dermatomyositis: a multicentre longitudinal cohort study. | Therapeutic cohort / case series; Anti-MDA5+ DM or DM-ILD | Comparative treatment exposure and longitudinal outcomes | mortality/prognosis; treatment response/control | Found no uniform prognostic advantage for triple-combination therapy in a multicenter longitudinal cohort. | Treatment-context evidence; selection and time-varying treatment were considered when interpreting outcomes. |
| 49 | Wu W et al. (2025). Effectiveness and safety of tofacitinib versus calcineurin inhibitor in interstitial lung disease secondary to anti-MDA5-positive dermatomyositis: a multicentre cohort study. | Therapeutic cohort / case series; Anti-MDA5+ DM or DM-ILD | Treatment exposure and clinical outcome | ILD phenotype/severity | Compared tofacitinib with calcineurin inhibitors using multicenter observational data and weighted survival analysis. | Treatment-context evidence; selection and time-varying treatment were considered when interpreting outcomes. |
| 50 | Chen Z et al. (2026). Distinct anti-MDA5 antibody trajectories associated with long-term outcomes in 6-month survivors of anti-MDA5 dermatomyositis with interstitial lung disease. | Clinical phenotype / outcome cohort; Anti-MDA5+ DM or DM-ILD | Functional or longitudinal autoantibody assessment | mortality/prognosis; ILD phenotype/severity | Identified anti-MDA5 antibody trajectories associated with long-term control among 6-month survivors. | Observational evidence interpreted in relation to setting, landmark, treatment era, and outcome definition. |
| 51 | Chen Z et al. (2017). HLA-DRB1 Alleles as Genetic Risk Factors for the Development of Anti-MDA5 Antibodies in Patients with Dermatomyositis. | Molecular / patient-derived mechanistic study; Han Chinese: PM n=70, DM n=104, and controls n=400 | Germline genetic association / sequencing | clinical or molecular phenotype | Associated HLA-DRB1 alleles with anti-MDA5 antibody development in a Han Chinese cohort. | Association or discovery evidence; assay standardization, sampling landmark, and independent replication were charted. |
| 52 | Guo L et al. (2023). WDFY4 polymorphisms in Chinese patients with anti-MDA5 dermatomyositis is associated with rapid progressive interstitial lung disease. | Molecular / patient-derived mechanistic study; Anti-MDA5+ DM n=254, anti-MDA5-negative DM n=53, antisynthetase syndrome n=72, and controls n=192 | Germline genetic association / sequencing | ILD phenotype/severity | Associated WDFY4 polymorphisms with RP-ILD in Chinese anti-MDA5+ DM. | Association or discovery evidence; assay standardization, sampling landmark, and independent replication were charted. |
| 53 | Yang X et al. (2025). The Prospective Registry of MyositIS (PROMIS): I. | Molecular / patient-derived mechanistic study; PROMIS: anti-MDA5+ DM n=231, anti-MDA5-negative DM n=127, disease controls n=1,468, and controls n=1,027 | Germline genetic association / sequencing | clinical or molecular phenotype | Nominated HLA-DQA1*06:01 as an anti-MDA5-associated susceptibility allele in PROMIS. | Association or discovery evidence; assay standardization, sampling landmark, and independent replication were charted. |
| 54 | Okamoto M et al. (2025). Gene variants of interferon induced with helicase C domain 1 in Japanese patients with Dermatomyositis-associated rapidly progressive interstitial lung disease: a genetic association study using whole-exome and Sanger sequencing. | Molecular / patient-derived mechanistic study; Japanese multicenter DM n=204 (ILD n=174; anti-MDA5+ n=62); WES discovery n=9 followed by targeted sequencing | Germline genetic association / sequencing | RP-ILD; ILD phenotype/severity | Identified candidate IFIH1 variants associated with dermatomyositis-related acute-onset ILD. | Association or discovery evidence; assay standardization, sampling landmark, and independent replication were charted. |
| 56 | Wang K et al. (2021). RNA-Containing Immune Complexes Formed by Anti-Melanoma Differentiation Associated Gene 5 Autoantibody Are Potent Inducers of IFN-α. | Molecular / patient-derived mechanistic study; Human DM/IIM cohorts or patient-derived material | Interferon-pathway measurement | clinical or molecular phenotype | Showed that anti-MDA5-RNA immune complexes induce interferon-alpha in vitro. | Mechanistic discovery evidence; sample size, treatment timing, or temporal ordering informs interpretation. |
| 57 | Coutant F et al. (2022). Monoclonal antibodies from B cells of patients with anti-MDA5 antibody-positive dermatomyositis directly stimulate interferon gamma production. | Molecular / patient-derived mechanistic study; Anti-MDA5+ DM or DM-ILD | Interferon-pathway measurement | clinical or molecular phenotype | Showed that patient-derived anti-MDA5 monoclonal antibodies can stimulate interferon-gamma production. | Mechanistic discovery evidence; sample size, treatment timing, or temporal ordering informs interpretation. |
| 58 | Li M et al. (2023). Type 1 interferon signature in peripheral blood mononuclear cells and monocytes of idiopathic inflammatory myopathy patients with different myositis-specific autoantibodies. | Molecular / patient-derived mechanistic study; RNA-seq in IIM n=23 (anti-MDA5+ n=9) and controls n=8, with validation cohorts | Interferon-pathway measurement | clinical or molecular phenotype | Localized the strongest type I interferon program in anti-MDA5+ IIM largely to monocytes. | Association or discovery evidence; assay standardization, sampling landmark, and independent replication were charted. |
| 59 | Wang Y et al. (2023). Transcriptomic profiling and longitudinal study reveal the relationship of anti-MDA5 titer and type I IFN signature in MDA5+ dermatomyositis. | Molecular / patient-derived mechanistic study; RNA-seq in patients n=13 and controls n=7; longitudinal cohort n=49 | Blood or tissue transcriptomics | clinical or molecular phenotype | Demonstrated distinct longitudinal kinetics of anti-MDA5 titers and the type I interferon signature. | Association or discovery evidence; assay standardization, sampling landmark, and independent replication were charted. |
| 60 | Liu Y et al. (2023). IFN-beta and EIF2AK2 are potential biomarkers for interstitial lung disease in anti-MDA5 positive dermatomyositis. | Biomarker / longitudinal cohort; Anti-MDA5+ DM or DM-ILD | Interferon-pathway measurement | ILD phenotype/severity | Nominated IFN-beta and EIF2AK2 as candidate markers of pulmonary involvement. | Association or discovery evidence; assay standardization, sampling landmark, and independent replication were charted. |
| 61 | Kokuzawa A et al. (2023). Potential role of type I interferon/IP-10 axis in the pathogenesis of anti-MDA5 antibody-positive dermatomyositis. | Molecular / patient-derived mechanistic study; Anti-MDA5+ DM or DM-ILD | Interferon-pathway measurement | pathobiology | Supported a type I interferon-IP-10 axis in anti-MDA5+ DM pathobiology. | Association or discovery evidence; assay standardization, sampling landmark, and independent replication were charted. |
| 62 | Fukada A et al. (2024). Prognostic Role of Interferon-λ3 in Anti-Melanoma Differentiation-Associated Gene 5-Positive Dermatomyositis-Associated Interstitial Lung Disease. | Molecular / patient-derived mechanistic study; PM/DM-ILD n=221 and controls n=38; anti-MDA5+ outcome cohort n=79 | Baseline serum IFN-λ3 with prognostic derivation/validation | Poor pulmonary outcome | Established serum IFN-lambda3 as a prognostic marker in derivation and validation cohorts. | Association or discovery evidence; assay standardization, sampling landmark, and independent replication were charted. |
| 63 | Yoshida A et al. (2025). Dysregulated type I/III interferon system in circulation from patients with anti-MDA5-positive dermatomyositis. | Molecular / patient-derived mechanistic study; Anti-MDA5+ DM n=53; circulating type I/III interferon profiling | Interferon-pathway measurement | clinical or molecular phenotype | Identified dysregulated and clinically distinct circulating type I/III interferon patterns. | Mechanistic discovery evidence; sample size, treatment timing, or temporal ordering informs interpretation. |
| 64 | Xue K et al. (2025). Transcriptomic signatures in different tissues of anti-MDA5 positive dermatomyositis: Uncovering genes with predictive potential for RP-ILD. | Molecular / patient-derived mechanistic study; Anti-MDA5+ DM or DM-ILD | Blood or tissue transcriptomics | RP-ILD | Mapped shared and tissue-specific transcriptomic programs and nominated candidate RP-ILD genes. | Association or discovery evidence; assay standardization, sampling landmark, and independent replication were charted. |
| 65 | Zhang M et al. (2026). Dysregulation of YTHDF2 promotes the over expression of interferon beta in anti-MDA5 antibody-positive dermatomyositis. | Molecular / patient-derived mechanistic study; Anti-MDA5+ DM or DM-ILD | Interferon-pathway measurement | clinical or molecular phenotype | Linked reduced monocyte YTHDF2 to excess interferon-beta expression. | Mechanistic discovery evidence; sample size, treatment timing, or temporal ordering informs interpretation. |
| 66 | Zhang X et al. (2026). Interferon-β Triggers Z-DNA Binding Protein 1-Mediated PANoptosis in T Cells of Anti-Melanoma Differentiation-Associated Protein 5 Antibody-Positive Dermatomyositis. | Molecular / patient-derived mechanistic study; Anti-MDA5+ DM or DM-ILD | Interferon-pathway measurement | clinical or molecular phenotype | Linked interferon-beta to ZBP1-mediated PANoptosis in T cells. | Mechanistic discovery evidence; sample size, treatment timing, or temporal ordering informs interpretation. |
| 67 | He J et al. (2024). Single-cell landscape of peripheral immune response in patients with anti-melanoma differentiation-associated gene 5 dermatomyositis. | Molecular / patient-derived mechanistic study; DM n=8, including anti-MDA5+ n=5; PBMC single-cell RNA sequencing | Single-cell transcriptomics and immune-cell states | pathobiology | Identified IFI27/IFIH1-high monocytes, plasma cells, and broader peripheral immune-state shifts. | Mechanistic discovery evidence; sample size, treatment timing, or temporal ordering informs interpretation. |
| 68 | Weng C et al. (2025). Single-cell sequencing reveals distinct peripheral immune responses in anti-MDA5 antibody positive dermatomyositis with rapidly progressive interstitial lung disease. | Molecular / patient-derived mechanistic study; RP-ILD n=4, non-RP-ILD ILD n=3, and controls n=3; PBMC single-cell RNA sequencing | Single-cell transcriptomics and immune-cell states | RP-ILD; ILD phenotype/severity; pathobiology | Associated RP-ILD with expanded monocytes, depleted T/NK cells, antiviral activation, and weaker antigen presentation. | Mechanistic discovery evidence; sample size, treatment timing, or temporal ordering informs interpretation. |
| 69 | Shi J et al. (2025). Monocyte-macrophage dynamics as key in disparate lung and peripheral immune responses in severe anti-melanoma differentiation-associated gene 5-positive dermatomyositis-related interstitial lung disease. | Molecular / patient-derived mechanistic study; Severe anti-MDA5+ DM-ILD n=5 with paired blood/BALF; orthogonal validation cohorts | Paired blood/BALF single-cell transcriptomics with flow-cytometric and Luminex validation | Severe pulmonary inflammation and remodeling | Traced circulating monocytes toward inflammatory and profibrotic lung-macrophage states. | Mechanistic discovery evidence; sample size, treatment timing, or temporal ordering informs interpretation. |
| 70 | Shao C et al. (2025). Exhaustion of NK cells and interferon activation in anti-MDA5(+) dermatomyositis are associated and determine the development of ILD. | Molecular / patient-derived mechanistic study; Anti-MDA5+ DM or DM-ILD | Interferon-pathway measurement | clinical or molecular phenotype | Linked NK-cell exhaustion and interferon activation to ILD development. | Association or discovery evidence; assay standardization, sampling landmark, and independent replication were charted. |
| 71 | Yang Y et al. (2026). IgG from anti-MDA5⁺ CADM patients impairs NK cell function via CD16 in RP-ILD. | Molecular / patient-derived mechanistic study; Clinically amyopathic DM / DM-ILD | Functional or longitudinal autoantibody assessment | RP-ILD | Showed that patient IgG impairs NK-cell function through CD16 and promotes macrophage activation. | Mechanistic discovery evidence; sample size, treatment timing, or temporal ordering informs interpretation. |
| 72 | Qian J et al. (2026). Reduced peripheral CD4+ memory T-cell counts mark a mortality-associated immunophenotype in anti-MDA5 dermatomyositis. | Molecular / patient-derived mechanistic study; Anti-MDA5+ DM n=64; 13 deaths by day 180; CD4+ memory profiling | Clinical or immunophenotypic clustering | mortality/prognosis | Associated reduced peripheral CD4+ memory T-cell counts with mortality. | Mechanistic discovery evidence; sample size, treatment timing, or temporal ordering informs interpretation. |
| 73 | Hensgens MPM et al. (2022). Clinical features and immune-related protein patterns of anti-MDA5 positive clinically amyopathic dermatomyositis Dutch patients. | Molecular / patient-derived mechanistic study; Dutch CADM n=20 (serum subset n=16) plus controls n=15 | Serum/plasma protein profiling | clinical or molecular phenotype | Defined a broad inflammatory and interferon-related serum-protein profile in Dutch anti-MDA5+ CADM. | Mechanistic discovery evidence; sample size, treatment timing, or temporal ordering informs interpretation. |
| 74 | Zhang Y et al. (2025). Shared and Distinctive Inflammation-Related Protein Profiling in Idiopathic Inflammatory Myopathy with/without Anti-MDA5 Autoantibodies. | Molecular / patient-derived mechanistic study; IIM n=77 (anti-MDA5+ n=34) plus controls n=53 | Serum/plasma protein profiling | clinical or molecular phenotype | Identified shared and anti-MDA5-specific inflammatory-protein patterns across IIM. | Association or discovery evidence; assay standardization, sampling landmark, and independent replication were charted. |
| 75 | Liang L et al. (2021). Aberrantly Expressed Galectin-9 Is Involved in the Immunopathogenesis of Anti-MDA5-Positive Dermatomyositis-Associated Interstitial Lung Disease. | Molecular / patient-derived mechanistic study; Anti-MDA5+ DM or DM-ILD | Serum galectin-9 measurement and mechanistic assays | ILD severity and immune-pathway activity | Linked elevated galectin-9 with anti-MDA5+ DM-ILD severity and immune activation. | Mechanistic discovery evidence; sample size, treatment timing, or temporal ordering informs interpretation. |
| 76 | Li T et al. (2025). Integrated metabolomic profiling identifies citrate as novel diagnostic biomarker for Anti-MDA5-Positive dermatomyositis. | Molecular / patient-derived mechanistic study; Active untreated IIM n=183 plus controls n=104; anti-MDA5+ discovery subgroup n=67 and independent validation | Metabolomic or metabolic-pathway profiling | clinical or molecular phenotype | Identified citrate and dimethylglycine within coordinated plasma and blood-cell metabolic programs. | Association or discovery evidence; assay standardization, sampling landmark, and independent replication were charted. |
| 77 | Xiao Y et al. (2026). Multiomics analysis uncovers subtype-specific mechanisms and biomarkers in idiopathic inflammatory myopathies. | Biomarker / longitudinal cohort; Idiopathic inflammatory myopathy cohorts | Within-patient multi-omics profiling across IIM subtypes | IIM subtype mechanisms and biomarkers | Demonstrated coordinated within-patient multi-omics across IIM subtypes and nominated subtype-specific biomarkers. | Association or discovery evidence; assay standardization, sampling landmark, and independent replication were charted. |
| 80 | Kitahara Y et al. (2026). Serum interferon-λ3 as a short-term biomarker of disease control in anti-MDA5-positive dermatomyositis-associated ILD. | Molecular / patient-derived mechanistic study; Anti-MDA5+ DM-ILD n=24; diagnosis and 1-month serum IFN-λ3 | Interferon-pathway measurement | treatment response/control | Associated failure of serum IFN-lambda3 to decline by 1 month with death or relapse. | Mechanistic discovery evidence; sample size, treatment timing, or temporal ordering informs interpretation. |
| 81 | Gono T et al. (2022). Antiviral proinflammatory phenotype of monocytes in anti-MDA5 antibody-associated interstitial lung disease. | Molecular / patient-derived mechanistic study; Anti-MDA5+ DM or DM-ILD | Monocyte phenotyping, transcriptomic, and functional immune profiling | Anti-MDA5-associated ILD inflammatory phenotype | Defined an antiviral, proinflammatory monocyte phenotype in anti-MDA5-associated ILD. | Association or discovery evidence; assay standardization, sampling landmark, and independent replication were charted. |
| 82 | Guo J et al. (2023). Risk Prediction for Rapidly Progressive Interstitial Lung Disease in Anti-MDA5-Positive Dermatomyositis: The CRAFT Model. | Prediction / stratification study; Anti-MDA5+ DM n=53; 21 RP-ILD events; baseline predictors | Clinical prediction or risk-stratification model | Incident RP-ILD | Developed the internally validated CRAFT score for incident RP-ILD. | Internal validation or derivation only; independent geographic validation remains the next test. |
| 83 | So J et al. (2022). Predictors of rapidly progressive interstitial lung disease and mortality in patients with autoantibodies against melanoma differentiation-associated protein 5 dermatomyositis. | Prediction / stratification study; Anti-MDA5+ DM n=116; 47 RP-ILD events; multicenter baseline cohort and small internal validation subset | FLAW clinical risk score | Incident RP-ILD and mortality | Developed the FLAW score for RP-ILD and identified separate baseline mortality predictors. | Internal validation or derivation only; independent geographic validation remains the next test. |
| 84 | Lian X et al. (2020). Mortality Risk Prediction in Amyopathic Dermatomyositis Associated With Interstitial Lung Disease: The FLAIR Model. | Prediction / stratification study; Amyopathic DM-ILD n=207; baseline 1-year mortality model | Clinical prediction or risk-stratification model | mortality/prognosis; ILD phenotype/severity | Developed the FLAIR model for 1-year mortality in amyopathic DM-ILD. | Internal validation or derivation only; independent geographic validation remains the next test. |
| 85 | Gono T et al. (2021). Risk Prediction Modeling Based on a Combination of Initial Serum Biomarker Levels in Polymyositis/Dermatomyositis-Associated Interstitial Lung Disease. | Prediction / stratification study; Incident PM/DM-ILD n=497 derivation and n=111 independent validation | MCK baseline mortality model | All-cause mortality | Developed and independently validated the MCK model for mortality in incident PM/DM-ILD. | Independent, geographic, or temporal validation reported; population and landmark remain relevant to transportability. |
| 86 | Gui X et al. (2022). Prediction model for the pretreatment evaluation of mortality risk in anti-melanoma differentiation-associated gene 5 antibody-positive dermatomyositis with interstitial lung disease. | Prediction / stratification study; Anti-MDA5+ DM-ILD n=89 discovery and n=38 temporal validation; pretreatment | Clinical prediction or risk-stratification model | mortality/prognosis; ILD phenotype/severity; treatment response/control | Developed and temporally validated a pretreatment mortality model for anti-MDA5+ DM-ILD. | Independent, geographic, or temporal validation reported; population and landmark remain relevant to transportability. |
| 87 | Niu Q et al. (2022). A New Predictive Model for the Prognosis of MDA5(+) DM-ILD. | Prediction / stratification study; Anti-MDA5+ DM-ILD n=63; 19 events; baseline prognostic model | Clinical prediction or risk-stratification model | mortality/prognosis | Developed a baseline prognostic model combining clinical, CT, and laboratory factors. | Internal validation or derivation only; independent geographic validation remains the next test. |
| 88 | Ouyang ZM et al. (2022). A Matrix Prediction Model for the 6-Month Mortality Risk in Patients With Anti-Melanoma Differentiation-Associated Protein-5-Positive Dermatomyositis. | Prediction / stratification study; Anti-MDA5+ DM n=40 derivation and n=29 external validation; baseline 6-month mortality model | Clinical prediction or risk-stratification model | mortality/prognosis | Developed and externally validated a three-factor matrix for 6-month mortality. | Independent, geographic, or temporal validation reported; population and landmark remain relevant to transportability. |
| 89 | Zong C et al. (2025). The FLATCAN Model: A Novel Score for Predicting Mortality Risk in Anti-Melanoma Differentiation-Associated Gene 5-Positive Dermatomyositis. | Prediction / stratification study; Anti-MDA5+ DM n=621; baseline mortality model | Clinical prediction or risk-stratification model | mortality/prognosis | Developed the FLATCAN time-to-event mortality score in a large anti-MDA5+ DM cohort. | Internal validation or derivation only; independent geographic validation remains the next test. |
| 90 | Xu W et al. (2021). A Computed Tomography Radiomics-Based Prediction Model on Interstitial Lung Disease in Anti-MDA5-Positive Dermatomyositis. | Prediction / stratification study; Anti-MDA5+ DM-ILD n=228 across training, test, temporal, and external cohorts | CT radiomics plus clinical survival model | 6-month all-cause mortality | Developed and externally validated a CT radiomics-plus-clinical model for 6-month mortality. | Independent, geographic, or temporal validation reported; population and landmark remain relevant to transportability. |
| 91 | Li Y et al. (2024). A nomogram based on clinical factors and CT radiomics for predicting anti-MDA5+ DM complicated by RP-ILD. | Prediction / stratification study; Anti-MDA5+ DM n=103; baseline clinical and CT-radiomic assessment | Clinical-radiomic RP-ILD nomogram | Incident RP-ILD | Developed a clinical-radiomic nomogram for incident RP-ILD and reported test-set performance. | Internal validation or derivation only; independent geographic validation remains the next test. |
| 92 | Zhang C et al. (2026). Semiquantitative HRCT scoring: a reliable predictor of rapidly progressive interstitial lung disease in patients with anti-MDA5 positive dermatomyositis. | Prediction / stratification study; Anti-MDA5+ DM n=67; baseline semiquantitative HRCT and clinical data | HRCT phenotype, score, or radiomics | RP-ILD; ILD phenotype/severity | Evaluated semiquantitative HRCT extent as an accessible RP-ILD predictor. | Internal validation or derivation only; independent geographic validation remains the next test. |
| 97 | Xu A et al. (2021). Prognostic values of anti-Ro52 antibodies in anti-MDA5-positive clinically amyopathic dermatomyositis associated with interstitial lung disease. | Biomarker / longitudinal cohort; Clinically amyopathic DM / DM-ILD | Candidate soluble or serological biomarker | ILD phenotype/severity | Associated anti-Ro52 positivity with adverse pulmonary outcomes in anti-MDA5+ CADM-ILD. | Association or discovery evidence; assay standardization, sampling landmark, and independent replication were charted. |
| 98 | Lv C et al. (2023). Coexistence of Anti-Ro52 Antibodies in Anti-MDA5 Antibody-Positive Dermatomyositis Is Highly Associated With Rapidly Progressive Interstitial Lung Disease and Mortality Risk. | Biomarker / longitudinal cohort; Anti-MDA5+ DM or DM-ILD | Candidate soluble or serological biomarker | RP-ILD; mortality/prognosis; ILD phenotype/severity | Associated coexisting anti-Ro52 antibodies with RP-ILD and mortality. | Association or discovery evidence; assay standardization, sampling landmark, and independent replication were charted. |
| 99 | Zhu Y et al. (2022). Serum Krebs von den Lungen-6 concentrations reflect severity of anti-melanoma differentiation-associated protein 5 antibody positive dermatomyositis associated interstitial lung disease. | Clinical phenotype / outcome cohort; Anti-MDA5+ DM or DM-ILD | Serum KL-6 measurement | ILD severity | Showed that serum KL-6 concentrations track anti-MDA5+ DM-ILD severity. | Observational evidence interpreted in relation to setting, landmark, treatment era, and outcome definition. |
| 100 | Gui X et al. (2021). Cytokeratin 19 fragment is associated with severity and poor prognosis of interstitial lung disease in anti-MDA5 antibody-positive dermatomyositis. | Biomarker / longitudinal cohort; Anti-MDA5+ DM or DM-ILD | Candidate soluble or serological biomarker | mortality/prognosis; ILD phenotype/severity | Associated CYFRA21-1 with ILD severity and poor prognosis. | Association or discovery evidence; assay standardization, sampling landmark, and independent replication were charted. |
| 101 | Shimizu T et al. (2021). IL-15 is a biomarker involved in the development of rapidly progressive interstitial lung disease complicated with polymyositis/dermatomyositis. | Biomarker / longitudinal cohort; PM/DM-ILD cohorts; anti-MDA5 subgroup where reported | Candidate soluble or serological biomarker | RP-ILD; ILD phenotype/severity | Identified IL-15 as a candidate biomarker for RP-ILD in PM/DM. | Association or discovery evidence; assay standardization, sampling landmark, and independent replication were charted. |
| 102 | Shi Y et al. (2024). Elevated serum B-cell activator factor levels predict rapid progressive interstitial lung disease in anti-melanoma differentiation associated protein 5 antibody positive dermatomyositis. | Biomarker / longitudinal cohort; Human DM/IIM cohorts or patient-derived material | Candidate soluble or serological biomarker | ILD phenotype/severity | Associated elevated serum BAFF with RP-ILD in anti-MDA5+ DM. | Association or discovery evidence; assay standardization, sampling landmark, and independent replication were charted. |
| 103 | Wu Y et al. (2025). Anti-MDA5 antibody IgG1 subtype is associated with rapidly progressive interstitial lung disease in anti-MDA5-positive dermatomyositis. | Clinical phenotype / outcome cohort; Anti-MDA5+ DM or DM-ILD | Anti-MDA5 IgG1-subclass assessment | Incident RP-ILD | Associated the anti-MDA5 IgG1 subtype with RP-ILD. | Observational evidence interpreted in relation to setting, landmark, treatment era, and outcome definition. |
| 104 | Xu YT et al. (2022). Evaluation and validation of the prognostic value of anti-MDA5 IgG subclasses in dermatomyositis-associated interstitial lung disease. | Clinical phenotype / outcome cohort; Anti-MDA5+ DM or DM-ILD | Functional or longitudinal autoantibody assessment | ILD phenotype/severity | Evaluated and validated the prognostic value of anti-MDA5 IgG subclasses. | Observational evidence interpreted in relation to setting, landmark, treatment era, and outcome definition. |
| 105 | Kogami M et al. (2023). Changes in anti-MDA5 antibody titres and serum cytokine levels before and after diagnosis of anti-MDA5 antibody-positive dermatomyositis. | Biomarker / longitudinal cohort; Anti-MDA5+ DM or DM-ILD | Functional or longitudinal autoantibody assessment | clinical or molecular phenotype | Characterized longitudinal anti-MDA5 titers and serum cytokines around diagnosis and treatment. | Association or discovery evidence; assay standardization, sampling landmark, and independent replication were charted. |
| 106 | Zhang Y et al. (2026). Serum ferritin trajectories predict mortality risk in anti-melanoma differentiation-associated protein 5 antibody-positive dermatomyositis: a longitudinal cohort study. | Clinical phenotype / outcome cohort; Anti-MDA5+ DM or DM-ILD | Serial serum-ferritin trajectory modeling | Mortality | Identified longitudinal ferritin trajectories that separated mortality risk. | Observational evidence interpreted in relation to setting, landmark, treatment era, and outcome definition. |
| 107 | Chen Z et al. (2019). Tofacitinib in Amyopathic Dermatomyositis-Associated Interstitial Lung Disease. | Therapeutic cohort / case series; Clinically amyopathic DM / DM-ILD | Treatment exposure and clinical outcome | ILD phenotype/severity | Reported tofacitinib use in amyopathic DM-associated ILD. | Treatment-context evidence; selection and time-varying treatment were considered when interpreting outcomes. |
| 109 | Bay P et al. (2022). Efficacy of plasma exchange in patients with anti-MDA5 rapidly progressive interstitial lung disease. | Therapeutic cohort / case series; Anti-MDA5+ DM or DM-ILD | Treatment exposure and clinical outcome | RP-ILD; ILD phenotype/severity; treatment response/control | Evaluated plasma exchange in anti-MDA5 RP-ILD. | Treatment-context evidence; selection and time-varying treatment were considered when interpreting outcomes. |
| 110 | Ge Y et al. (2021). Anti-melanoma differentiation-associated gene 5 (MDA5) antibody-positive dermatomyositis responds to rituximab therapy. | Therapeutic cohort / case series; Anti-MDA5+ DM or DM-ILD | Treatment exposure and clinical outcome | treatment response/control | Reported clinical responses to rituximab in anti-MDA5+ dermatomyositis. | Treatment-context evidence; selection and time-varying treatment were considered when interpreting outcomes. |

**Table S1B. Secondary evidence syntheses (n=15)**

| **Ref.** | **Evidence source** | **Design / population or material** | **Index / data type** | **Pulmonary or clinical endpoint** | **Principal finding or contribution** | **Appraisal note** |
| --- | --- | --- | --- | --- | --- | --- |
| 1 | Lundberg IE et al. (2021). Idiopathic inflammatory myopathies. | Narrative or focused review; Published human evidence | Structured or narrative synthesis of published evidence | clinical or molecular phenotype | Synthesized published evidence on idiopathic inflammatory myopathies; used for context and not counted as independent primary replication. | Potential overlap with included primary reports; used as contextual synthesis only. |
| 2 | Lu X et al. (2024). Anti-MDA5 antibody-positive dermatomyositis: pathogenesis and clinical progress. | Narrative or focused review; Published human evidence | Structured or narrative synthesis of published evidence | pathobiology | Synthesized published evidence on anti-MDA5 antibody-positive dermatomyositis: pathogenesis and clinical progress; used for context and not counted as independent primary replication. | Potential overlap with included primary reports; used as contextual synthesis only. |
| 3 | Yoshida T et al. (2025). Anti-Melanoma Differentiation-Associated Gene 5 Antibody Positive Dermatomyositis: Recent Progress in Pathophysiology and Treatment. | Narrative or focused review; Published human evidence | Structured or narrative synthesis of published evidence | treatment response/control | Synthesized published evidence on anti-Melanoma Differentiation-Associated Gene 5 Antibody Positive Dermatomyositis: Recent Progress in Pathophysiology and Treatment; used for context and not counted as independent primary replication. | Potential overlap with included primary reports; used as contextual synthesis only. |
| 6 | Nombel A et al. (2021). Dermatomyositis With Anti-MDA5 Antibodies: Bioclinical Features, Pathogenesis and Emerging Therapies. | Narrative or focused review; Published human evidence | Structured or narrative synthesis of published evidence | pathobiology | Synthesized published evidence on dermatomyositis With Anti-MDA5 Antibodies: Bioclinical Features, Pathogenesis and Emerging Therapies; used for context and not counted as independent primary replication. | Potential overlap with included primary reports; used as contextual synthesis only. |
| 7 | Wu W et al. (2021). Interstitial Lung Disease in Anti-MDA5 Positive Dermatomyositis. | Narrative or focused review; Published human evidence | Structured or narrative synthesis of published evidence | ILD phenotype/severity | Synthesized published evidence on interstitial Lung Disease in Anti-MDA5 Positive Dermatomyositis; used for context and not counted as independent primary replication. | Potential overlap with included primary reports; used as contextual synthesis only. |
| 11 | Sehgal S et al. (2025). Idiopathic inflammatory myopathies related lung disease in adults. | Narrative or focused review; Published human evidence | Structured or narrative synthesis of published evidence | ILD phenotype/severity | Synthesized published evidence on idiopathic inflammatory myopathies related lung disease in adults; used for context and not counted as independent primary replication. | Potential overlap with included primary reports; used as contextual synthesis only. |
| 12 | Xie H et al. (2023). Risk factors for mortality in patients with anti-MDA5 antibody-positive dermatomyositis: A meta-analysis and systematic review. | Systematic review / meta-analysis; Published human evidence | Structured or narrative synthesis of published evidence | mortality/prognosis | Synthesized published evidence on risk factors for mortality in patients with anti-MDA5 antibody-positive dermatomyositis: A meta-analysis and systematic review; used for context and not counted as independent primary replication. | Potential overlap with included primary reports; used as contextual synthesis only. |
| 13 | Yang Y et al. (2025). Risk factors for mortality in anti-MDA5 antibody-positive dermatomyositis with interstitial lung disease: a systematic review and meta-analysis. | Systematic review / meta-analysis; Published human evidence | Structured or narrative synthesis of published evidence | mortality/prognosis; ILD phenotype/severity | Synthesized published evidence on risk factors for mortality in anti-MDA5 antibody-positive dermatomyositis with interstitial lung disease: a systematic review and meta-analysis; used for context and not counted as independent primary replication. | Potential overlap with included primary reports; used as contextual synthesis only. |
| 14 | Liu T et al. (2026). Predictors of rapidly progressive interstitial lung disease in anti-MDA5 dermatomyositis: a meta-analysis. | Systematic review / meta-analysis; Published human evidence | Structured or narrative synthesis of published evidence | RP-ILD; ILD phenotype/severity | Synthesized published evidence on predictors of rapidly progressive interstitial lung disease in anti-MDA5 dermatomyositis: a meta-analysis; used for context and not counted as independent primary replication. | Potential overlap with included primary reports; used as contextual synthesis only. |
| 15 | Li X et al. (2022). Roles of biomarkers in anti-MDA5-positive dermatomyositis, associated interstitial lung disease, and rapidly progressive interstitial lung disease. | Narrative or focused review; Published human evidence | Structured or narrative synthesis of published evidence | RP-ILD; ILD phenotype/severity | Synthesized published evidence on roles of biomarkers in anti-MDA5-positive dermatomyositis, associated interstitial lung disease, and rapidly progressive interstitial lung disease; used for context and not counted as independent primary replication. | Potential overlap with included primary reports; used as contextual synthesis only. |
| 28 | Lattarulo S et al. (2025). Anti-MDA5+ dermatomyositis following SARS-COV-2 infections: a systematic review. | Systematic review / meta-analysis; Published human evidence | Structured or narrative synthesis of published evidence | clinical or molecular phenotype | Synthesized published evidence on anti-MDA5+ dermatomyositis following SARS-COV-2 infections: a systematic review; used for context and not counted as independent primary replication. | Potential overlap with included primary reports; used as contextual synthesis only. |
| 29 | Liu S et al. (2026). When viral infections meet the anti-MDA5 antibody-positive dermatomyositis. | Narrative or focused review; Published human evidence | Structured or narrative synthesis of published evidence | clinical or molecular phenotype | Synthesized published evidence on when viral infections meet the anti-MDA5 antibody-positive dermatomyositis; used for context and not counted as independent primary replication. | Potential overlap with included primary reports; used as contextual synthesis only. |
| 30 | Li H et al. (2026). Influencing factors for rapidly progressive interstitial lung disease in patients with anti-MDA5 antibody-positive dermatomyositis: a systematic review and meta-analysis. | Systematic review / meta-analysis; Published human evidence | Structured or narrative synthesis of published evidence | RP-ILD; ILD phenotype/severity | Synthesized published evidence on influencing factors for rapidly progressive interstitial lung disease in patients with anti-MDA5 antibody-positive dermatomyositis: a systematic review and meta-analysis; used for context and not counted as independent primary replication. | Potential overlap with included primary reports; used as contextual synthesis only. |
| 31 | Izuka S et al. (2025). The role of monocytes and macrophages in idiopathic inflammatory myopathies: insights into pathogenesis and potential targets. | Narrative or focused review; Published human evidence | Structured or narrative synthesis of published evidence | pathobiology | Synthesized published evidence on the role of monocytes and macrophages in idiopathic inflammatory myopathies: insights into pathogenesis and potential targets; used for context and not counted as independent primary replication. | Potential overlap with included primary reports; used as contextual synthesis only. |
| 108 | Wang Y et al. (2024). The efficacy and safety of tofacitinib in anti-melanoma differentiation-associated gene 5 antibody positive dermatomyositis associated interstitial lung disease: a systematic review and meta-analysis. | Systematic review / meta-analysis; Published human evidence | Structured or narrative synthesis of published evidence | ILD phenotype/severity; treatment response/control | Synthesized published evidence on the efficacy and safety of tofacitinib in anti-melanoma differentiation-associated gene 5 antibody positive dermatomyositis associated interstitial lung disease: a systematic review and meta-analysis; used for context and not counted as independent primary replication. | Potential overlap with included primary reports; used as contextual synthesis only. |
